# Discovery of 2-(4-Substituted-piperidin/piperazine-1-yl)-*N*-(5-cyclopropyl-1*H*-pyrazol-3-yl)-quinazoline-2,4-diamines as PAK4 Inhibitors with Potent A549 Cell Proliferation, Migration, and Invasion Inhibition Activity

**DOI:** 10.3390/molecules23020417

**Published:** 2018-02-14

**Authors:** Tianxiao Wu, Yu Pang, Jing Guo, Wenbo Yin, Mingyue Zhu, Chenzhou Hao, Kai Wang, Jian Wang, Dongmei Zhao, Maosheng Cheng

**Affiliations:** Key Laboratory of Structure-Based Drug Design & Discovery of Ministry of Education, Shenyang Pharmaceutical University, Shenyang 110016, China; 15330802221@163.com (T.W.); pangyu038@163.com (Y.P.); guojingspu@163.com (J.G.); yinwenbo1994@163.com (W.Y.); 13555832736@163.com (M.Z.); spuhcz@163.com (C.H.); 15702442831@163.com (K.W.); Jianwang@email.com (J.W.); mscheng@263.net (M.C.)

**Keywords:** p21-activated kinase, PAK4 inhibitor, 2,4-diaminoquinazoline, anticancer

## Abstract

A series of novel 2,4-diaminoquinazoline derivatives were designed, synthesized, and evaluated as p21-activated kinase 4 (PAK4) inhibitors. All compounds showed significant inhibitory activity against PAK4 (half-maximal inhibitory concentration IC_50_ < 1 μM). Among them, compounds **8d** and **9c** demonstrated the most potent inhibitory activity against PAK4 (IC_50_ = 0.060 μM and 0.068 μM, respectively). Furthermore, we observed that compounds **8d** and **9c** displayed potent antiproliferative activity against the A549 cell line and inhibited cell cycle distribution, migration, and invasion of this cell line. In addition, molecular docking analysis was performed to predict the possible binding mode of compound **8d**. This series of compounds has the potential for further development as PAK4 inhibitors for anticancer activity.

## 1. Introduction

The p21-activated kinases (PAKs), which are members of the serine/threonine protein kinases, are activated by the Rho family of small guanosine-5′-triphosphate (GTP)-binding proteins Rac and Cdc42 [1,2]. Based on sequence and structural homology, six different PAKs members are classified into two groups (PAK1–3 in group I, and PAK4–6 in group II) [3,4,5]. PAKs are key effectors of the Rho family GTPases Rac and Cdc42, and their expression and activity are thought to play critical roles in cellular function, including promoting cell growth, inhibiting cell apoptosis, and regulating cytoskeleton functions [1,6,7,8,9]. Focus is currently shifting from group I PAKs to group II PAKs [10], because of the acute cardiovascular toxicity resulting from the inhibition of PAK2 [11] and the unique biological function of PAK4. It was reported in 2017 that although PAK1 and PAK4 are highly expressed in HeLa cervical cancer cells, only PAK4 knockdown attenuates expression of HIF-1α in hypoxia [12]. In addition, recent studies have shown that PAK4 played an indispensable part in effective migration and invasion of prostate, ovarian, pancreatic, and glioma cancer cell lines [13,14]. These studies confirmed the scientific rationale for further development of PAK4 inhibitors as anticancer agents.

**Figure 1:**
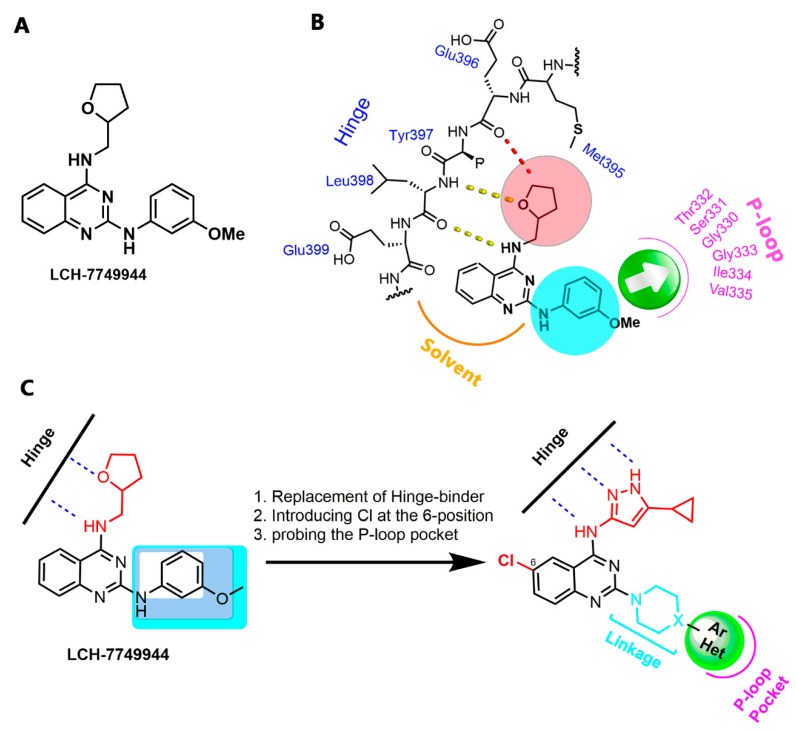
(**A**) Chemical structure of LCH-7749944; (**B**) Binding mode of LCH-7749944 within the PAK4 binding site; (**C**) Design strategy of the target compounds.

In our prior study [15], *N*^2^-(3-methoxyphenyl)-*N*^4^-((tetrahydrofuran-2-yl)methyl)quinazoline-2,4-diamine (LCH-7749944) was identified as a novel and potent inhibitor of PAK4 (Figure 1A). LCH-7749944 moderately inhibited PAK4 activity with a half-maximal inhibitory concentration (IC_50_) of 14.93 μM and effectively suppressed the proliferation, migration, and invasion of human gastric cancer cells through downregulation of several PAK4-dependent pathways. 

Molecular docking studies showed that the furan fragment at the C4-position of LCH-7749944 can form two hydrogen bonding interactions with PAK4 hinge residues Tyr397 and Leu398, and the C2-position substituent extended to the P-loop pocket (Figure 1B). Given that LCH-7749944 was not tightly filled with the P-loop pocket and that larger cavities were still left unoccupied, it may result in increased potency that incorporating functional motifs of suitable length and H-bonding potential at 2-position of quinazoline ring offered access to amino acid residues in the P-loop pocket. The hinge-binder fragment was also the focus of our investigation, by replacing the original group with a 3-amino-5-cyclopropyl-pyrazole fragment, which can form the classic three-point donor-acceptor-donor hydrogen bond interactions with the PAK4 hinge residues Glu396 and Leu398 (Figure 1C). On the basis of the above strategy, we designed and synthesized 19 novel 2,4-diaminoquinazoline derivatives.

## 2. Results and Discussion

### 2.1. Chemistry

**Scheme 1:**
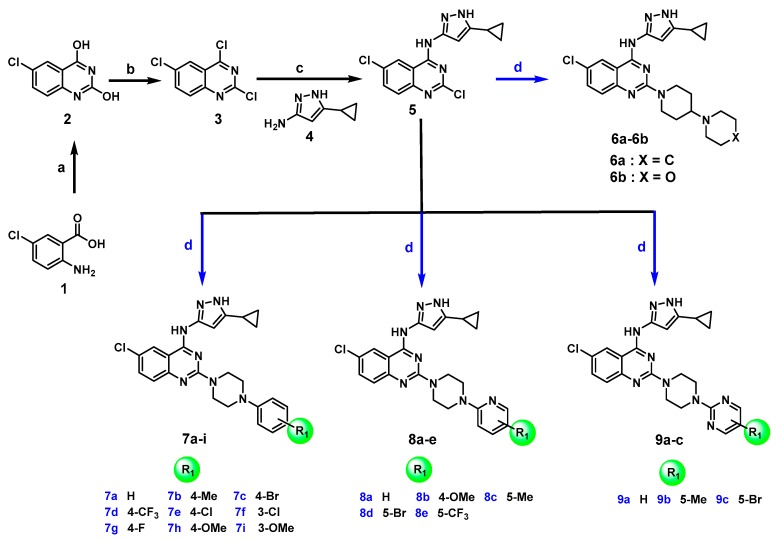
Synthesis of target compounds **6a**,**b**, **7a**–**i**, **8a**–**e**, **9a**–**c**. *Reagent and conditions*: (**a**) Urea, 200 °C, 98%; (**b**) POCl_3_, *N*,*N*-diethylaniline, reflux, 80.2%; (**c**) *N*,*N*-Diisopropylethylamine (DIEA), **4**, dimethylformamide (DMF), 0 °C, 74.6%. (**d**) DIEA, KI, DMF, appropriate substituted piperidine/piperazine, 65 °C, overnight, 57.2–70.6%.

Synthesis of the designed 2,4-diaminoquinazoline derivatives **6a**,**b**, **7a**–**i**, **8a**–**e**, **9a**–**c** employed 2,6-dichloro-*N*-(5-cyclopropyl-1*H*-pyrazol-3-yl)-quinazolin-4-amine (**5**) as the key intermediate. This intermediate was obtained from 2-amino-5-chlorobenzoic acid (**1**) through the synthetic methodology depicted in Scheme 1. Treatment of **1** with urea at 200 °C provided a good yield of **2**. Then, the resulting intermediate **2** was subjected to chlorination with phosphoryl trichloride to produce **3**. Under basic conditions, the 4-position chlorine of **3** was selectively substituted with 3-amino-5-cyclopropylpyrazole to give the key intermediate **5** [16]. Finally, the chlorine at the 2-position of **5** was substituted with available piperazine or piperidine derivatives in the presence of potassium iodide to provide the target compounds **6a**,**b**, **7a**–**i**, **8a**–**e**, **9a**–**c**. These compounds were confirmed by proton nuclear magnetic resonance (^1^H-NMR), carbon-13 nuclear magnetic resonance (^13^C-NMR) and, High-resolution accurate mass determinations (HRMSs). (Supplementary Materials)

### 2.2. In Vitro Activity against PAK4 Kinase and Structure-Activity Relationships

As mentioned earlier, docking studies indicated that extending the length of the molecules may be helpful in increasing their activity. Hence, we fixed the quinazoline scaffold as well as the 4-substituted 3-amino-5-cyclopropylpyrazole moiety and introduced piperazines or piperidines at the 2-position to increase the length of the molecule. At the end of this linker, a hydrophobic aromatic ring or alicyclic ring was introduced to explore the P-loop region. In total, 19 compounds against PAK4 were determined by using the homogeneous time-resolved fluorescence (HTRF) kinase assay.

The enzyme inhibitory activities are summarized in Table 1. Introducing an alicyclic ring at the end of the linkage to increase the length of the molecules (compounds **6a**,**b**) resulted in better inhibitory activity than LCH-7749944. Replacing the alicyclic ring with a phenyl ring (compounds **7a**–**i**) increased PAK4 potency, except for **7a**, indicating that flat geometric configuration was beneficial. Phenyl rings containing substituents (compounds **7b**–**i**) exhibited higher kinase inhibition than non-substituted derivatives (compound **7a**), and the *para*-substituted phenyl analogs (**7e**,**g**) showed higher enzyme activity than the *meta*-substituted analogs **7f**,**h**. Furthermore, we examined the effects of different substituents at the same position (*para*-substituted phenyl ring derivatives **7b**–**e**,**i**). The activities were in the order 4-Br > 4-Cl > 4-F > 4-Me > 4-CF_3_. Analog **7c** displayed strong inhibition against PAK4, suggesting that bromine atoms had a moderate spatial structure to occupy the hydrophobic cavity of the P-loop pocket. Upon investigation of other aromatic rings, better activity was seen when the N-4 of piperazine was substituted with a pyridine (compounds **8a**,**c**,**d**) and pyrimidine (compounds **9a−c**) groups compared with phenyl substituted derivatives **7a−c**. A possible explanation is that the nitrogen atom of the pyridine and pyrimidine group form a stronger hydrogen-bonding interaction with the hydrophobic region of PAK4. Br-substituted pyridine **8d** (IC_50_ = 0.060 µM) and pyrimidine **9c** (IC_50_ = 0.068 µM), like Br-substituted phenyl **7c**, showed better potency than other analogs **8a**,**c** and **9a**,**b**. 

### 2.3. Effects of Compounds ***8d*** and ***9c*** on Cell Proliferation

Based on the enzymatic assay, compounds **8d** and **9c** were selected for cellular assay on the A549 cell line, in which PAK4 has been found to be overexpressed. Meanwhile, the tumor cell line HT1080, whose growth is not dependent on PAK4, was used to test the potential off-target effects of the potent PAK4 inhibitors. Both **8d** and **9c** demonstrated potent inhibition against the A549 cell line (IC_50_ = 4.685 μM, and 4.751 μM, respectively), and negligible activity was observed for HT1080 (Table 2).

### 2.4. Effects of Compound ***8d*** on Cell Cycle Progression, Cell Migration, and Cell Invasion

**Figure 2:**
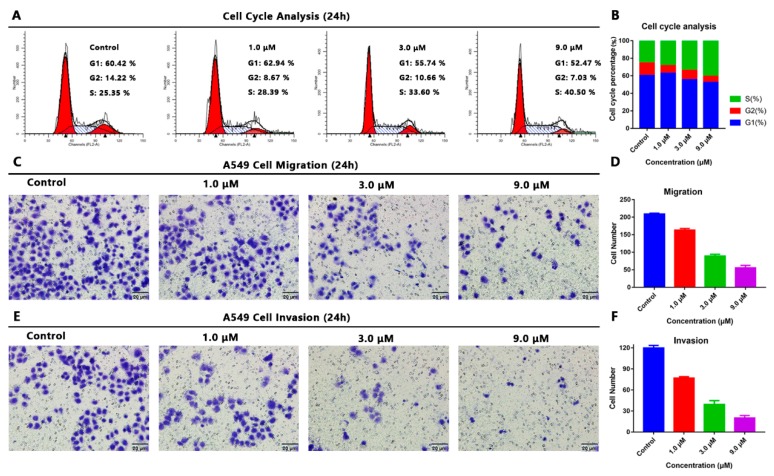
(**A**) Compound **8d** induced S cell cycle arrest of A549 cells. A549 cells were harvested after treatment with various concentrations of compound **8d** for 24 h; (**B**) Quantitative analysis of cell cycle; (**C**,**E**) Compound **8d** inhibited migration and invasion of A549 cells in transwell assays. The images were captured using phase contrast microscopy after 24 h of treatment. Scale bar, 20 μM; (**D**,**F**) Quantitative analysis of migration and invasion.

We next examined the effects of compound **8d** on the cycle progression of A549. Cells were treated with compound **8d** at varying concentrations (1.0, 3.0, 9.0 μM) and vehicle (dimethyl sulfoxide−DMSO) for 24 h, and subjected to flow cytometry analysis to determine the distribution of cells in various phases of the cell cycle. Compound **8d** was found to induce a dose-dependent decrease in the percentage of cells in the G1 phase and an increase in the S phase compared with the vehicle control (Figure 2A,B), indicating that compound **8d** arrested A549 cells at the S phase of the cell cycle. PAK4 activity is involved in cytoskeleton dynamics and cancer cell migration and invasion; therefore, the inhibitory effect of **8d** on A549 cell migration and invasion were analyzed. As displayed in Figure 2C–F, **8d** decreased the migration and invasion potential of the A549 cells in a dose-dependent manner. These results demonstrate that **8d** efficiently exhibits antimetastatic potential against cancer cells.

### 2.5. Binding Mode Analysis

**Figure 3:**
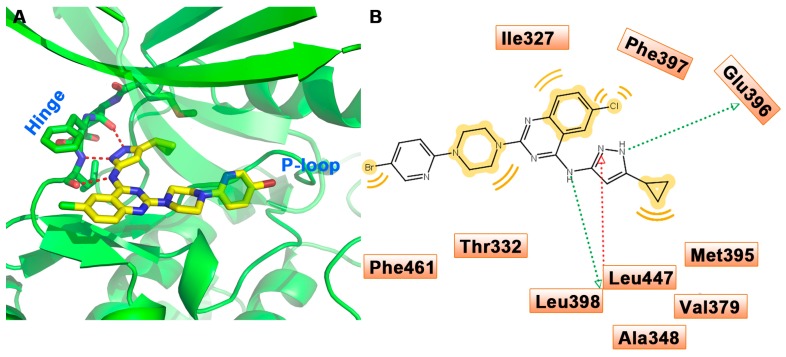
Model of compound **8d** bound to PAK4, generated by energy minimization of the elaborated structure of **8d** within the PAK4 protein using the MMFF94 force field within LigandScout. (**A**) Detailed interactions with the protein residues. Hydrogen bonds are rendered as dotted red lines; (**B**) 2D interaction diagram showing compound **8d** docking pose interactions with the key amino acids in the PAK4 active site by LigandScout.

To investigate potential binding modes, compound **8d** was docked into the ATP-binding site of PAK4 (Protein Data Bank ID: 2X4Z) using Autodock 4.2 (Figure 3). 

Compound **8d** binds to the PAK4 catalytic domain in the ATP binding site and makes multiple contacts with the hinge region through hydrogen-bond interactions with the pyrazole motif and the amine linker to the quinazoline ring. The piperidine ring of **8d** adopted a twist-boat conformation. This led to projection of the equatorial pyridine substituent along a more direct vector to the P-loop, which sat within a hydrophobic environment created by the P-loop backbone and the side chain of Phe461.

## 3. Experimental

### 3.1. Chemicals and Instruments 

Unless otherwise noted, all materials were obtained from commercially available sources and were used without purification. Thin-layer chromatography (TLC) was performed on silica gel plates with F-254 indicator (Shanghai jingke industrial Co. Ltd., Shanghai, China) and visualized by UV light. Proton nuclear magnetic resonance (^1^H-NMR, 400 MHz) and carbon-13 nuclear magnetic resonance (^13^C-NMR, 151 MHz) spectra were recorded using a Bruker 400 MHz NMR spectrometer (Bruker, Karlsruhe, Germany) in DMSO-*d*_6_, and the chemical shifts were recorded in parts per million downfield from tetramethylsilane. High-resolution accurate mass determinations (HRMSs) for all final target compounds were obtained on a Bruker Micromass Time of Flight mass spectrometer (Bruker Bioscience, Billerica, MA, USA) equipped with electrospray ionization (ESI). Column chromatography was performed on silica gel (200–300 mesh) purchased from Qingdao Haiyang Chemical Co. Ltd. (Qingdao, China).

### 3.2. Synthesis

*6-Chl.oroquinazoline-2,4-diol* (**2**). This compound was synthesized according to previously reported methods [17].

*2,4,6-Trichloroquinazoline* (**3**). This compound was synthesized according to previously reported methods [18].

*2,6-Dichloro-N-(5-cyclopropyl-1H-pyrazol-3-yl)quinazolin-4-amine* (**5**). A solution of 5-cyclopropyl-1*H*-pyrazol-3-amine (0.267 g, 2.17 mmol) and 2,4,6-trichloroquinazoline (0.432 g, 2.17 mmol) in dimethylformamide (DMF) (10 mL) was treated with EtN(i-Pr)_2_ (0.49 mL, 2.82 mmol) and stirred for 4 h at 0 °C. The reaction mixture was poured over ice water. The solid was collected by filtration and dried in air to provide compound **5** without further purification [16]. The yield was 74.6%.

#### General Procedure for Synthesis of Compounds **6a**,**b**, **7a**–**i**, **8a**–**e**, **9a**–**c**

The corresponding piperazine or piperidine (1.1 mmol), KI (0.18 g, 1.1 mmol), and DIEA (0.21 mL, 1.3 mmol) was added to a solution of compound **5** (0.32 g, 1 mmol) in DMF (10 mL). The reaction mixture was stirred at 60 °C overnight. After completion of the reaction, the reaction mixture was cooled to room temperature and poured over ice water. The solid was collected by filtration and purified by chromatography on silica gel (dichloromethane/methanol, 30:1) to give the title compounds **6a**,**b**, **7a**–**i**, **8a**–**e**, **9a**–**c** in moderate yields.

*([1,4′-Bipiperidin]-1′-yl)-6-chloro-N-(5-cyclopropyl-1H-pyrazol-3-yl)quinazolin-4-amine* (**6a**). White solid, yield 65.3%, m.p.: 208.3–209.6 °C. ^1^H-NMR (DMSO-*d*_6_) δ 12.22 (s, 1H), 10.10 (s, 1H), 8.52 (s, 1H), 7.52 (dd, *J* = 8.9, 1.9 Hz, 1H), 7.30 (d, *J* = 8.9 Hz, 1H), 6.35 (s, 1H), 4.78 (d, *J* = 12.7 Hz, 2H), 3.17 (d, *J* = 5.2 Hz, 1H), 2.83 (t, *J* = 12.1 Hz, 2H), 2.48~2.40 (m, 4H), 1.97~1.86 (m, 1H), 1.76 (d, *J* = 11.5 Hz, 2H), 1.49~1.44 (m, 4H), 1.40~1.32 (m, 4H), 0.99~0.94 (m, 2H), 0.7~0.66 (m, 2H). ^13^C-NMR (DMSO-*d*_6_) δ 158.79, 157.14, 151.58, 147.96, 145.91, 133.28, 127.47, 124.81, 123.03, 111.36, 94.55, 62.72, 50.13, 43.79, 28.03, 26.57, 25.05, 8.42, 7.30. HRMS calcd. for C_24_H_30_ClN_7_, [M + H]^+^, 452.2331; found 452.2331.

*5-Chloro-N-(5-cyclopropyl-1H-pyrazol-3-yl)-2-(4-morpholinopiperidin-1-yl)quinazolin-4-amine* (**6b**). White solid, yield 67.4%, m.p.: 214.3–216.6 °C. ^1^H-NMR (DMSO-*d*_6_) δ 12.23 (s, 1H), 10.11 (s, 1H), 8.53 (s, 1H), 7.53 (dd, *J* = 8.9, 1.8 Hz, 1H), 7.30 (d, *J* = 8.9 Hz, 1H), 6.35 (s, 1H), 4.75 (d, *J* = 13.0 Hz, 2H), 3.57–3.55 (m, 4H), 2.89 (t, *J* = 12.0 Hz, 2H), 2.49~2.43 (m, 4H), 1.98~1.89 (m, 1H), 1.84 (d, *J* = 10.9 Hz, 2H), 1.38–1.28 (m, 2H), 0.97 (d, *J* = 7.3 Hz, 2H), 0.70~0.66 (m, 2H). ^13^C-NMR (DMSO-*d*_6_) δ 158.80, 157.18, 151.56, 147.93, 145.90, 133.31, 127.48, 124.85, 123.04, 111.39, 94.60, 67.05, 62.10, 49.85, 43.39, 28.25, 8.44, 7.30. HRMS calcd. for C_23_H_28_ClN_7_O, [M + H]^+^, 454.2119; found 454.2119.

*6-Chloro-N-(5-cyclopropyl-1H-pyrazol-3-yl)-2-(4-phenylpiperazin-1-yl)quinazolin-4-amine* (**7a**). White solid, yield 69.0%, m.p.: 239.5–240.8 °C. ^1^H-NMR (DMSO-*d*_6_) δ 12.97 (s, 1H), 10.26 (s, 1H), 8.55 (s, 1H), 7.71 (d, *J* = 7.5 Hz, 2H), 7.52 (d, *J* = 8.5 Hz, 1H), 7.44 (t, *J* = 7.4 Hz, 2H), 7.31 (d, *J* = 4.2 Hz, 2H), 7.16 (t, *J* = 7.9 Hz, 2H), 7.06 (s, 1H), 6.94 (d, *J* = 8.1 Hz, 2H), 6.73 (t, *J* = 7.2 Hz, 1H), 3.96~3.87 (m, 4H), 3.22~3.06 (m, 4H). ^13^C-NMR (DMSO-*d*_6_) δ 158.98, 157.25, 151.60, 151.34, 147.84, 133.42, 129.41, 127.59, 152.21, 123.12, 119.62, 116.34, 111.64, 94.91, 48.90, 44.02, 8.49, 7.39. HRMS calcd. for C_24_H_24_ClN_7_, [M + H]^+^, 446.1859; found 446.1859.

*6-Chloro-N-(5-cyclopropyl-1H-pyrazol-3-yl)-2-(4-(p-tolyl)piperazin-1-yl)quinazolin-4-amine* (**7b**). White solid, yield 67.5%, m.p.: 204.9–207.5 °C. ^1^H-NMR (DMSO-*d*_6_) δ 12.24 (s, 1H), 10.16 (s, 1H), 8.57 (s, 1H), 7.57 (dd, *J* = 1.84, 9.00 Hz, 1H), 7.35 (d, *J* = 9.04 Hz, 1H), 7.05 (d, *J* = 8.40 Hz, 2H), 6.92 (d, *J* = 8.56 Hz, 2H), 6.39 (s, 1H), 3.94–3.91 (m, 4H), 3.16–3.14 (m, 4H), 2.22 (s, 3H), 1.97~1.93 (m, 1H), 0.99~0.97 (m, 2H), 0.73~0.72 (m, 2H). ^13^C-NMR (DMSO-*d*_6_) δ 158.92, 157.23, 151.24, 149.53, 147.75, 145.95, 133.47, 129.86, 128.52, 127.52, 125.26, 123.12, 116.67, 111.62, 94.85, 52.50, 49.42, 45.92, 44.09, 20.54, 8.99, 8.47, 7.65. HRMS calcd. for C_24_H_24_ClN_7_, [M + H]^+^, 460.2011; found 460.2011.

*2-(4-(4-Bromophenyl)piperazin-1-yl)-6-chloro-N-(5-cyclopropyl-1H-pyrazol-3-yl)quinazolin-4-amine* (**7c**). White solid, yield 59.2%, m.p.: 248.7–251.9 °C. ^1^H-NMR (DMSO-*d*_6_) δ 12.23 (s, 1H), 10.16 (s, 1H), 8.57 (s, 1H), 7.57 (dd, *J* = 8.8, 1.6 Hz, 1H), 7.37 (t, *J* = 9.0 Hz, 3H), 6.98 (d, *J* = 9.1 Hz, 2H), 6.39 (d, *J* = 0.5 Hz, 1H), 3.92~3.94 (m, 4H), 3.25~3.22 (m, 4H), 1.96~1.94 (m, 1H), 0.99~0.97 (m, 2H), 0.74~0.73 (m, 2H). ^13^C-NMR (DMSO-*d*_6_) δ 158.93, 157.24, 151.29, 150.69, 133.45, 131.95, 127.58, 125.24, 118.16, 111.63, 110.69, 48.45, 43.83, 8.48, 7.39. HRMS calcd. for C_24_H_23_BrClN_7_, [M + H]^+^, 526.0969; found 526.0969.

*6-Chloro-N-(5-cyclopropyl-1H-pyrazol-3-yl)-2-(4-(4-(trifluoromethyl)phenyl)piperazin-1-yl)quinazolin-4-amine* (**7d**). White solid, yield 67.4%, m.p.: 256.4–257.8 °C. ^1^H-NMR (DMSO-*d*_6_) δ 12.24 (s, 1H), 10.18 (s, 1H), 8.58 (d, *J* = 1.1 Hz, 1H), 7.57 (dd, *J* = 9.1, 1.9 Hz, 1H), 7.54 (d, *J* = 8.8 Hz, 2H), 7.36 (d, *J* = 8.9 Hz, 1H), 7.14 (d, *J* = 8.8 Hz, 2H), 6.41 (s, 1H), 4.04~3.85 (m, 4H), 3.49~3.37 (m, 4H), 2.08~1.86 (m, 1H), 1.09~0.92 (m, 2H), 0.86~0.66 (m, 2H). ^13^C-NMR (DMSO-*d*_6_) δ 158.95, 157.28, 153.72, 151.30, 147.82, 145.94, 133.45, 127.58, 126.66, 126.62, 125.28, 123.14, 118.55, 118.23, 114.82, 111.68, 94.93, 47.36, 43.69, 8.49, 7.40. HRMS calcd. for C_25_H_23_ClF_3_N_7_, [M + H]^+^, 514.1744; found 514.1744.

*6-Chloro-2-(4-(4-chlorophenyl)piperazin-1-yl)-N-(5-cyclopropyl-1H-pyrazol-3-yl)quinazolin-4-amine* (**7e**). White solid, yield 61.6%, m.p.: 250–251.8 °C. ^1^H-NMR (DMSO-*d*_6_) δ 12.22 (s, 1H), 10.17 (s, 1H), 8.56 (s, 1H), 7.57 (dd, *J* = 8.9, 2.2 Hz, 1H), 7.35 (d, *J* = 8.9 Hz, 1H), 7.26 (d, *J* = 9.0 Hz, 2H), 7.03 (d, *J* = 9.1 Hz, 2H), 6.38 (d, *J* = 0.8 Hz, 1H)., 4.19~3.67 (m, 4H), 3.29~3.08 (m, 4H), 1.97~1.93 (m, 1H), 0.99~0.97 (m, 2H), 0.77~0.70 (m, 2H). ^13^C-NMR (DMSO-*d*_6_) δ 158.96, 157.26, 151.31, 150.38, 145.92, 133.44, 129.10, 127.59, 122.25, 123.06, 117.73, 111.65, 94.89, 48.61, 43.86, 8.48, 7.39. HRMS calcd. for C_24_H_23_Cl_2_N_7_, [M + H]^+^, 480.1471; found 480.1471.

*6-Chloro-2-(4-(3-chlorophenyl)piperazin-1-yl)-N-(5-cyclopropyl-1H-pyrazol-3-yl)quinazolin-4-amine* (**7f**). White solid, yield 57.2%, m.p.: 229.9–232.6 °C. ^1^H-NMR (DMSO-*d*_6_) δ 12.23 (s, 1H), 10.17 (s, 1H), 8.57 (s, 1H), 7.57 (dd, *J* = 8.8, 1.7 Hz, 1H), 7.36 (d, *J* = 8.9 Hz, 1H), 7.24 (t, *J* = 8.1 Hz, 1H), 7.03 (s, 1H), 6.98 (dd, *J* = 8.4, 1.8 Hz, 1H), 6.81 (dd, *J* = 7.8, 1.2 Hz, 1H), 6.40 (s, 1H), 3.92~3.91 (m, 4H), 3.31~3.16 (m, 4H), 1.99~1.90 (m, 1H), 1.01~0.95 (m, 2H), 0.76~0.72 (m, 2H). ^13^C-NMR (DMSO-*d*_6_) δ 158.95, 157.25, 152.79, 151.31, 147.81, 145.93, 134.30, 133.44, 130.88, 127.59, 125.26, 123.12, 118.73, 115.37, 114.46, 111.66, 94.95, 48.20, 43.83, 8.49, 7.42. HRMS calcd. for C_24_H_23_Cl_2_N_7_, [M + H]^+^, 480.1465; found 480.1461.

*6-Chloro-N-(5-cyclopropyl-1H-pyrazol-3-yl)-2-(4-(4-methoxyphenyl)piperazin-1-yl)quinazolin-4-amine* (**7g**). White solid, yield 70.1%, m.p.: 237.1–239.5 °C. ^1^H-NMR (DMSO-*d*_6_) δ 12.23 (s, 1H), 10.16 (s, 1H), 8.57 (d, *J* = 1.5 Hz, 1H), 7.56 (dd, *J* = 8.9, 2.0 Hz, 1H), 7.35 (d, *J* = 8.9 Hz, 1H), 6.98 (d, *J* = 9.1 Hz, 2H), 6.84 (d, *J* = 9.1 Hz, 2H), 6.39 (d, *J* = 0.4 Hz, 1H), 3.93~3.92 (m, 4H), 3.15~3.01 (m, 4H), 2.04~1.88 (m, 1H), 0.99~0.97 (m, 2H), 0.73~0.72 (m, 2H). ^13^C-NMR (DMSO-*d*_6_) δ 158.97, 157.25, 153.69, 151.36, 145.98, 133.42, 127.57, 125.18, 123.11, 118.49, 114.73, 111.62, 94.87, 55.65, 50.43, 44.16, 8.46, 7.36. HRMS calcd. for C_25_H_26_ClN_7_O, [M + H]^+^, 476.1966; found 476.1966.

*6-Chloro-N-(5-cyclopropyl-1H-pyrazol-3-yl)-2-(4-(3-methoxyphenyl)piperazin-1-yl)quinazolin-4-amine* (**7h**). White solid, yield 70.5%, m.p.: 199.9–201.8 °C. ^1^H-NMR (DMSO-*d*_6_) δ 12.22 (s, 1H), 10.14 (s, 1H), 8.56 (s, 1H), 7.56 (d, *J* = 8.8 Hz, 1H), 7.35 (d, *J* = 8.9 Hz, 1H), 7.13 (t, *J* = 8.2 Hz, 1H), 6.60 (dd, *J* = 8.2, 1.6 Hz, 1H), 6.53 (s, 1H), 6.40 (dd, *J* = 7.9, 1.8 Hz, 2H), 4.10~3.84 (m, 4H), 3.73 (s, 3H), 3.28~3.10 (m, 4H), 2.00~1.84 (m, 1H), 0.99~0.96 (m, 2H), 0.79~0.52 (m, 2H). ^13^C-NMR (DMSO-*d*_6_) δ 160.68, 158.97, 157.26, 152.97, 151.34, 147.85, 145.90, 133.41, 130.09, 127.57, 125.21, 123.13, 111.64, 108.96, 104.88, 102.47, 94.96, 55.36, 48.85, 43.99, 8.48, 7.41. HRMS calcd. for C_25_H_26_ClN_7_O, [M + H]^+^, 476.1992; found 476.1992.

*6-Chloro-N-(5-cyclopropyl-1H-pyrazol-3-yl)-2-(4-(4-fluorophenyl)piperazin-1-yl)quinazolin-4-amine* (**7i**). White solid, yield 66.2%, m.p.: 228.8–231 °C. ^1^H-NMR (DMSO-*d*_6_) δ 12.24 (s, 1H), 10.16 (s, 1H), 8.57 (d, *J* = 0.9 Hz, 1H), 7.57 (dd, *J* = 9.1, 1.7 Hz, 1H), 7.35 (d, *J* = 8.9 Hz, 1H), 7.14~6.98 (m, 4H), 6.39 (s, 1H), 3.99~3.88 (m, 4H), 3.23~3.11 (m, 4H), 2.06~1.87 (m, 1H), 1.02~0.94 (m, 2H), 0.78~0.69 (m, 2H). ^13^C-NMR (DMSO-*d*_6_) δ 158.96, 157.27, 151.33, 148.53, 147.82, 145.92, 145.09, 133.44, 127.58, 125.22, 123.12, 118.24, 118.17, 115.87, 115.66, 111.64, 94.90, 49.72, 44.03, 8.48, 7.37. HRMS calcd. for C_24_H_23_ClFN_7_, [M + H]^+^, 464.1763; found 464.1763.

*6-Chloro-N-(5-cyclopropyl-1H-pyrazol-3-yl)-2-(4-(pyridin-2-yl)piperazin-1-yl)quinazolin-4-amine* (**8a**). White solid, yield 59.3%, m.p.: 184.4–186.7 °C. ^1^H-NMR (DMSO-*d*_6_) δ 12.23 (s, 1H), 10.13 (d, *J* = 22.7 Hz, 1H), 8.56 (s, 1H), 8.14 (dd, *J* = 4.8, 1.4 Hz, 1H), 7.56~7.54 (m 2H), 7.35 (d, *J* = 8.9 Hz, 1H), 6.89 (d, *J* = 8.6 Hz, 1H), 6.66 (dd, *J* = 6.9, 5.0 Hz, 1H), 6.40 (s, 1H), 3.99~3.77 (m, 4H), 3.67~3.52 (m, 4H), 1.97~1.92 (m, 1H), 0.99~0.97 (m, 2H), 0.78~0.68 (m, 2H). ^13^C-NMR (DMSO-*d*_6_) δ 159.45, 159.02, 157.22, 151.34, 148.03, 138.02, 133.45, 127.57, 125.20, 123.10, 113.58, 111.63, 107.73, 94.81, 44.96, 43.81, 8.48, 7.42. HRMS calcd. for C_23_H_23_ClN_8_, [M + H]^+^, 447.1808; found 447.1808.

*6-Chloro-N-(5-cyclopropyl-1H-pyrazol-3-yl)-2-(4-(4-methoxypyridin-2-yl)piperazin-1-yl)quinazolin-4-amine* (**8b**). White solid, yield 68.8%, m.p.: 210.3–211.6 °C. ^1^H-NMR (DMSO-*d*_6_) δ 12.24 (s, 1H), 10.17 (s, 1H), 8.57 (s, 1H), 7.97 (d, *J* = 5.7 Hz, 1H), 7.57 (dd, *J* = 8.7, 1.9 Hz, 1H), 7.36 (d, *J* = 8.9 Hz, 1H), 6.41 (s, 1H), 6.37 (d, *J* = 1.7 Hz, 1H), 6.33 (dd, *J* = 5.7, 2.0 Hz, 1H), 3.91~3.87 (m, 4H), 3.80 (s, 3H), 3.62~3.57 (m, 4H), 2.00~1.92 (m, 1H), 1.0~0.98 (m, 2H), 0.75~0.72 (m, 2H). ^13^C-NMR (DMSO-*d*_6_) δ 167.40, 161.34, 159.02, 157.23, 151.33, 149.09, 147.90, 145.95, 133.44, 127.57, 125.20, 123.12, 111.63, 101.90, 94.92, 92.11, 55.49, 45.17, 43.82, 8.47, 7.38. HRMS calcd. for C_24_H_25_ClN_8_O, [M + H]^+^, 477.1914; found 477.1914.

*6-Chloro-N-(5-cyclopropyl-1H-pyrazol-3-yl)-2-(4-(5-methylpyridin-2-yl)piperazin-1-yl)quinazolin-4-amine* (**8c**). White solid, yield 70.3%, m.p.: 263–265.4 °C. ^1^H-NMR (DMSO-*d*_6_) δ 12.24 (s, 1H), 10.16 (s, 1H), 8.57 (s, 1H), 8.00 (s, 1H), 7.57 (dd, *J* = 8.9, 1.5 Hz, 1H), 7.42 (dd, *J* = 8.6, 1.9 Hz, 1H), 7.36 (d, *J* = 9.0 Hz, 1H), 6.85 (d, *J* = 8.7 Hz, 1H), 6.41 (s, 1H), 3.92~3.87 (m, 4H), 3.57~3.50 (m, 4H), 2.16 (s, 3H), 2.02~1.91 (m, 1H), 1.0~0.99 (m, 2H), 0.74~0.73 (m, 2H). ^13^C-NMR (DMSO-*d*_6_) δ 159.02, 158.04, 157.21, 151.33, 147.60, 138.82, 133.43, 127.56, 125.19, 123.12, 123.10, 122.16, 111.62, 107.64, 94.82, 45.44, 43.81, 1734, 8.47, 7.39. HRMS calcd. for C_24_H_25_ClN_8_, [M + H]^+^, 461.2023; found 461.2023.

*2-(4-(5-Bromopyridin-2-yl)piperazin-1-yl)-6-chloro-N-(5-cyclopropyl-1H-pyrazol-3-yl)quinazolin-4-amine* (**8d**). White solid, yield 66.9%, m.p.: 270.5–272.8 °C. ^1^H-NMR (DMSO-*d*_6_) δ 12.24 (s, 1H), 10.18 (s, 1H), 8.57 (d, *J* = 1.9 Hz, 1H), 8.21 (d, *J* = 2.5 Hz, 1H), 7.73 (dd, *J* = 9.1, 2.5 Hz, 1H), 7.57 (dd, *J* = 9.1, 2.2 Hz, 1H), 7.36 (d, *J* = 8.9 Hz, 1H), 6.93 (d, *J* = 9.2 Hz, 1H), 6.40 (d, *J* = 2.0 Hz, 1H), 3.96~3.83 (m, 4H), 3.71~3.53 (m, 4H), 2.02~1.91 (m, 1H), 1.01~0.98 (m, 2H), 0.74~0.73 (m, 2H). ^13^C-NMR (DMSO-*d*_6_) δ 158.98, 158.10, 157.24, 151.32, 148.22, 145.94, 140.17, 133.44, 127.57, 125.24, 123.13, 111.65, 109.71, 107.21, 94.87, 44.90, 43.65, 8.48, 7.38. HRMS calcd. for C_23_H_22_BrClN_8_, [M + H]^+^, 527.0894; found 527.0894.

*6-Chloro-N-(5-cyclopropyl-1H-pyrazol-3-yl)-2-(4-(5-(trifluoromethyl)pyridin-2-yl)piperazin-1-yl)quinazol-in-4-amine* (**8e**). White solid, yield 63.7%, m.p.: 247.6–259.6 °C. ^1^H-NMR (DMSO-*d*_6_) δ 12.24 (s, 1H), 10.19 (s, 1H), 8.58 (d, *J* = 1.9 Hz, 1H), 8.46 (s, 1H), 7.84 (dd, *J* = 9.1, 2.5 Hz, 1H), 7.57 (dd, *J* = 9.0, 2.0 Hz, 1H), 7.36 (d, *J* = 8.9 Hz, 1H), 7.04 (d, *J* = 9.0 Hz, 1H), 6.41 (d, *J* = 1.4 Hz, 1H), 4.02~3.86 (m, 4H), 3.78–3.77 (m, 4H), 2.09~1.85 (m, 1H), 1.01~0.98 (m, 2H), 0.77~0.72 (m, 2H). ^13^C-NMR (DMSO-*d*_6_) δ 160.63, 158.92, 157.23, 151.29, 145.74, 134.96, 133.44, 127.56, 126.70, 125.26, 123.13, 113.90, 113.59, 106.84, 94.87, 44.44, 43.63, 8.47, 7.38. HRMS calcd. for C_24_H_22_ClF_3_N_8_, [M + H]^+^, 515.1678; found 515.1678.

*6-Chloro-N-(5-cyclopropyl-1H-pyrazol-3-yl)-2-(4-(pyrimidin-2-yl)piperazin-1-yl)quinazolin-4-amine* (**9a**). White solid, yield 67.2%, mp: 249.3–250.5 °C. ^1^H-NMR (DMSO-*d*_6_) δ ppm 12.25 (s, 1H), 10.19 (s, 1H), 8.56 (s, 1H),8.41 (d, *J* = 4.68 Hz, 2H), 7.57 (dd, *J* = 1.92, 8.96 Hz, 1H), 7.36 (d, *J* = 8.88 Hz, 1H), 6.67 (t, *J* = 4.68 Hz, 1H), 6.39 (s, 1H), 3.89~3.84 (m, 8H), 2.00~1.92 (m, 1H), 1.00~0.98 (m, 2H), 0.74~0.70 (m, 2H). ^13^C-NMR (DMSO-*d*_6_) δ 161.72, 158.98, 158.44, 157.22, 157.15, 151.36, 147.43, 133.47, 127.55, 125.24, 123.11, 111.63, 110.73, 94.71, 43.85, 43.66, 8.46, 7.43. HRMS calcd for C_22_H_22_ClN_9_, [M + H]^+^, 448.1860; found 448.1760.

*6-Chloro-N-(5-cyclopropyl-1H-pyrazol-3-yl)-2-(4-(5-methylpyrimidin-2-yl)piperazin-1-yl)quinazolin-4-amine* (**9b**). White solid, yield 66.5%, m.p.: 262.8–264.7 °C. ^1^H-NMR (DMSO-*d*_6_) δ 12.25 (s, 1H), 10.18 (s, 1H), 8.57 (d, *J* = 1.8 Hz, 1H)., 8.27 (s, 2H), 7.56 (dd, *J* = 8.9, 2.0 Hz, 1H), 7.35 (d, *J* = 8.9 Hz, 1H), 6.40 (d, *J* = 1.5 Hz, 1H), 3.87~3.85 (m, 4H), 3.83~3.77 (m, 4H), 2.10 (s, 3H), 2.01~1.91 (m, 1H), 1.01–0.98 (m, 2H), 0.73~0.71 (m, 2H). ^13^C-NMR (DMSO-*d*_6_) δ 160.71, 159.01, 158.22, 157.22, 151.35, 147.84, 145.97, 133.42, 127.53, 125.19, 123.13, 118.91, 111.64, 94.83, 43.96, 43.84, 14.54, 8.47, 7.33. HRMS calcd. for C_23_H_24_ClN_9_, [M + H]^+^, 462.1926; found 462.1926.

*2-(4-(5-Bromopyrimidin-2-yl)piperazin-1-yl)-6-chloro-N-(5-cyclopropyl-1H-pyrazol-3-yl)quinazolin-4-amine* (**9c**). White solid, yield 70.6%, m.p.: 280.6–282.7 °C. ^1^H-NMR (DMSO-*d*_6_) δ 12.24 (s, 1H), 10.19 (s, 1H), 8.57 (d, *J* = 0.5 Hz, 1H), 8.51 (s, 2H), 7.57 (dd, *J* = 8.9, 1.7 Hz, 1H), 7.36 (d, *J* = 8.9 Hz, 1H), 6.39 (s, 1H), 3.92~3.87 (m, 4H), 3.85~3.81 (m, 4H), 2.02~1.91 (m, 1H), 1.03~0.93 (m, 2H), 0.73~0.71 (m, 2H). ^13^C-NMR (DMSO-*d*_6_) δ 160.02, 158.93, 158.48, 157.23, 155.87, 151.32, 145.99, 133.45, 127.56, 125.27, 123.11, 111.65, 105.99, 94.82, 43.93, 43.68, 8.46, 7.40. HRMS calcd. for C_22_H_21_BrClN_9_, [M + H]^+^, 528.0849; found 528.0849.

### 3.3. PAK4 HTRF Assays

STK Substrate 1-, 2-, or 3-biotin (1 vial of 500 µg lyophilized), streptavidin-XL665 (1 vial of 3 mg lyophilized), STK Antibody-Cryptate (1 vial of 20,000 tests lyophilized), 5× enzymatic buffer (1 vial of 50 mL liquid 5×), and HTRF detection buffer (1 vial of 200 mL liquid 1×) were purchased from Cis-Bio International (Tecan Infinite F200, Männedorf, Switzerland). PAK4, ATP (Sigma A7699), DTT (Sigma D0632), and MgCl_2_ (Sigma M1028) were purchased from Sigma-Aldrich (St. Louis, MO, USA).

First, PAK4, STK Substrate 1-, 2-, or 3-biotin, and ATP were diluted with kinase buffer (5 mM MgCl_2_, 1 mM DTT, 1 mM 5× buffer (HEPES 250 mM (pH 7.0), NaN_3_ 0.1%, BSA 0.05%, orthovanadate 0.5 mM), H_2_O) to prepare a working solution. In a typical Nunclon 384 flat-bottom white polystyrene catalog plate assay (Thermo Fisher Scientific, Waltham, MA, USA) each well contained the following reagents: 2 µL PAK4 or PAK1 (4.1 µg/µL), 2 µL STK Substrate 1-, 2-, or 3-biotin (1 µmol/L), 2 µL ATP (2.08 µmol/L), and 4 µL test compound in kinase buffer were added to give a total assay volume of 10 µL/well. Blank values were determined by the inclusion of 2 µL STK Substrate 1-, 2-, or 3-biotin (1 µmol/L), 2 µL ATP (2.08 µmol/L), and 6 µL kinase buffer. After mixing, the reaction mixture was incubated at room temperature for 50 min. Then 5 µL Streptavidin-XL665 in EDTA diluted with detection buffer and 5 µL STK Antibody-Cryptate in EDTA diluted with detection buffer were added. After mixing, the reaction mixture was incubated at room temperature for 50 min. 

Fluorescence were determined using a Infinite F500 multimodal plate reader (Tecan Infinite F200) with excitation at 340 nm and simultaneous signal collection at 670 and 612 nm. The time-resolved settings were set at 150 µs for the delay and 500 µs integration time. Date were calculated using the equation 1000× (670 nm/612 nm). Data were processed by using GraphPad Prism 6 (GraphPad Software Inc., San Diego, CA, USA) to obtain IC_50_.

### 3.4. Cell Proliferation Assay

The human pulmonary carcinoma cell line A549 and the human fibrosarcoma cell line HT1080 were cultured in RPMI-1640 medium containing 10% fetal bovine serum (FBS), at 37 °C in a humidified atmosphere containing 5% CO_2._

The in vitro antiproliferative activity of some of the target compounds was determined by the MTT assay (Amresco, Seattle, WA, USA). A suspension of 100 µL human cancer cells (5 × 10^4^/mL) was plated in a 96-well cell culture plate and incubated with 5% CO_2_ at 37 °C for 24 h. Then cells were exposed to various concentrations of compounds and further cultured for 24 h. After that, MTT was added and absorbance was measured at 490 nm using a EL × 800 microplate reader (BioTek, Winooski, VT, USA). Dose-response curves were plotted to determine the IC_50_ values using GraphPad Prism 5.0. Each IC_50_ value was expressed as mean ± standard deviation.

### 3.5. Cell Cycle Analysis by Flow Cytometry

Cells were seeded in a 6-well plate overnight and treated with different concentrations of compound **8d** the following day. After 24 h, the cells were collected by EDTA-free trypsinization, centrifuged at 2000 rpm for 5 min, washed with PBS, and fixed in 70% ethanol at 4 °C for 2 h. The cells were then put in a water bath, and 100 µL RNase A was added at 37 °C for 30 min. Finally, 400 µL PI was added, they were incubated in the dark for 30 min at 4 °C, and analyzed using flow cytometry (FACS Calibur, Becton-Dickinson, Franklin Lakes, NJ, USA). The data were analyzed using Flowjo software (FlowJo Vx 10.0, Ashland, Covington, KY, USA).

### 3.6. Cell Migration and Invasion Assay

Cell migration assays were evaluated in transwell chambers (Corning Incorporated, Corning, NY, USA). Cell invasion assays were evaluated in Matrigel invasion chambers (Becton-Dickinson). First, 0.5 × 10^5^ tumor cells were plated in the top chamber with RPMI-1640 medium without FBS. Test compound was added to the bottom chamber with 500 µL medium containing 20% FBS. After incubation for 24 h at 37 °C, the cells were fixed in 100% methanol and stained with 0.1% crystal violet, then washed with PBS; the cells that had not migrated from the top surface of the filters were removed with cotton. Migrated cells were quantified by counting them in three randomly selected fields on the diameter under a microscope at 200× magnification 

### 3.7. Molecular Docking Study

X-ray protein structure of PAK4 obtained from the Protein Data Bank (ID: 2X4Z) was prepared with AutoDockTools (AutoDock 4.2, The Scripps Research Institute, La Jolla, CA, USA). Both the native ligand and the indicated compounds were built using CORINA Classic (CORINA Version 4.1.0, Altamira, LLC, Nürnberg, Germany). The size of the box was set to 60 × 60 × 60 units in number of grid points, and grid spacing = 0.375 Å centered in native ligand using AutoGrid4. Docking was performed using the Lamarckian genetic algorithm in AutoDock4. Each docking experiment was performed 10 times, yielding 10 docked conformations, and the most favorable pose of **8d** was displayed.

## 4. Conclusions

In conclusion, a series of novel 2,4-diaminoquinazoline derivatives was designed and synthesized, and preliminary inhibitory activity on PAK4 was evaluated. Among 19 derivatives, MTT and flow cytometry studies suggested that **8d** and **9c** showed strong antiproliferative activity by inhibiting the transition of cells from the G1 phase to the S phase, and transwell assay demonstrated that **8d** strongly inhibited migration and invasion of A549 cell line. Finally, the molecular docking study demonstrated possible binding modes for the interactions between compound **8d** and PAK4. These suggest that compound **8d** could act as a potential PAK4 inhibitor to be used for further optimization.

## Figures and Tables

**Table 1:** IC_50_ values (µM) of the synthesized compounds against PAK4.

Compound	PAK4	Compound	PAK4	Compound	PAK4
	IC_50_^a^		IC_50_		IC_50_
**6a**	0.44	**7f**	0.49	**8d**	0.060
**6b**	0.56	**7g**	0.30	**8e**	0.22
**7a**	0.85	**7h**	0.69	**9a**	0.38
**7b**	0.24	**7i**	0.20	**9b**	0.11
**7c**	0.10	**8a**	0.31	**9c**	0.068
**7d**	0.65	**8b**	0.38	PF-3758309 ^b^	0.016
**7e**	0.16	**8c**	0.087		

^a^ IC_50_ values are calculated based on homogeneous time-resolved fluorescence (HTRF) assay; ^b^ PF-3758309 was used as a positive control.

**Table 2:** IC_50_^a^ (μM) of potent compounds on cell proliferation.

Compound	PAK4	Cells	
		A549	HT1080
**8d**	0.060	4.685	21.519
**9c**	0.068	4.751	39.303

^a^ IC_50_: concentration of the compound displaying 50% cell growth inhibition after 24 h of drug exposure, as determined by MTT assay.

## Supplementary Materials

The following are available online.
