# Supplementary material for: Discovery of 2-(4-Substituted-piperidin/piperazine-1-yl)-N-(5-cyclopropyl-1H-pyrazol-3-yl)-quinazoline-2,4-diamines as PAK4 Inhibitors with Potent A549 Cell Proliferation, Migration, and Invasion Inhibition Activity
Source: Molecules. 2018 Feb 14;23(2):417. doi: 10.3390/molecules23020417 (PMC6100240; doi:10.3390/molecules23020417)

Discovery of 2-(4-Substituted-piperidin/piperazine-1-yl)-N-(5-cyclopropyl-1H-pyrazol-3-yl)-Quinazoline-2,4-Diamines as PAK4 Inhibitors that Showed Potent A549 Cell Proliferation, Migration, and Invasion Inhibition

Tianxiao Wu, Yu Pang, Jing Guo, Wenbo Yin, Mingyue Zhu, Chenzhou Hao, Kai Wang, Jian Wang, Dongmei Zhao*, Maosheng Cheng

Key Laboratory of Structure-Based Drug Design & Discovery of Ministry of Education, Shenyang Pharmaceutical University, Shenyang 110016, China; 15330802221@163.com (T. W.); pangyu038@163.com (Y. P.); guojingspu@163.com (J. G.); yinwenbo1994@163.com (W. Y.); 13555832736@163.com (M. Z.); spuhcz@163.com (C. H.); 15702442831@163.com (K. W.); Jianwang@email.com (J. W.); mscheng@263.net (M. C.).
* Correspondence: medchemzhao@163.com; Tel.: +86-24-4352-0219

Content: ^{1}H NMR, ^{13}C NMR, HRMS for all target compounds reported in this study.

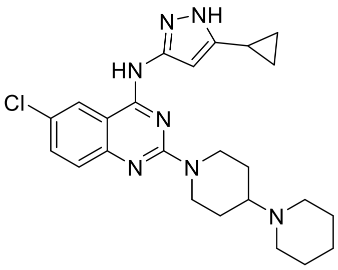
N–NH
HN
Cl
N
N
N
N

Compound **6a**

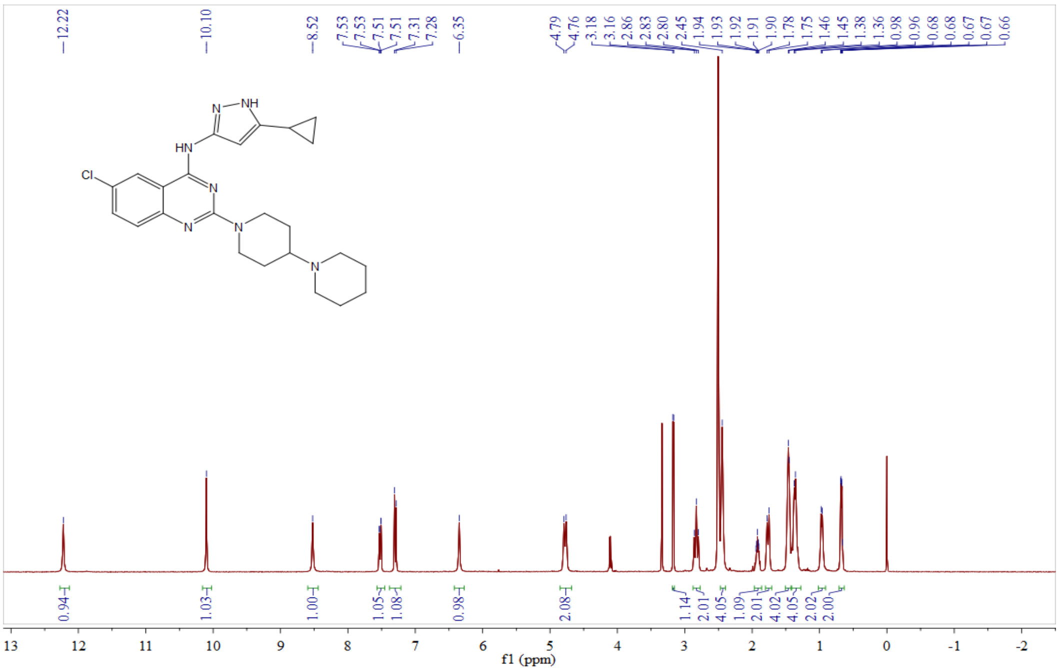
12.22
10.10
8.52
7.53
7.53
7.51
7.51
7.31
7.28
6.35
4.79
4.76
3.18
3.16
2.86
2.83
2.80
2.45
1.94
1.93
1.92
1.91
1.90
1.78
1.75
1.46
1.45
1.38
1.36
0.98
0.96
0.68
0.68
0.67
0.67
0.66
0.94
1.03
1.00
1.05
1.08
0.98
2.08
1.14
2.01
4.05
1.09
2.01
4.02
4.05
2.02
2.00
f1 (ppm)

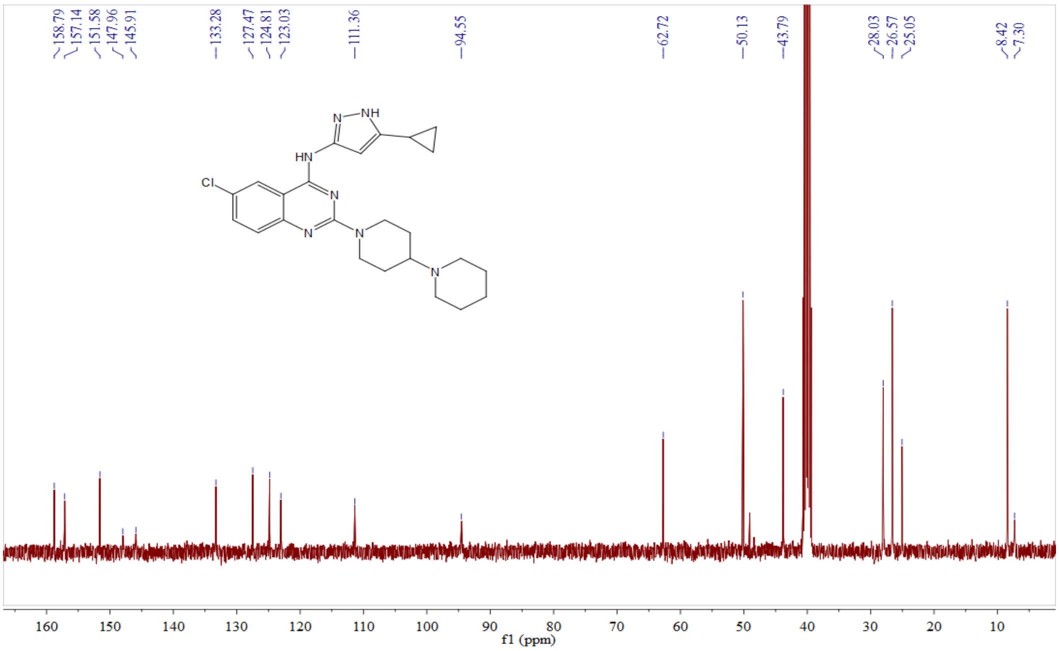
158.79
157.14
151.58
147.96
145.91
133.28
127.47
124.81
123.03
111.36
94.55
62.72
50.13
43.79
28.03
26.57
25.05
8.42
7.30
f1 (ppm)

User Spectra

Fragmentor Voltage	Collision Energy	Ionization Mode
200	0	ESI

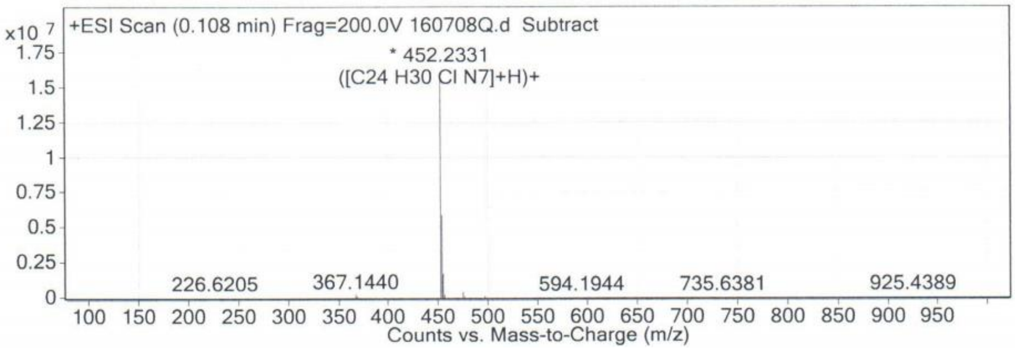

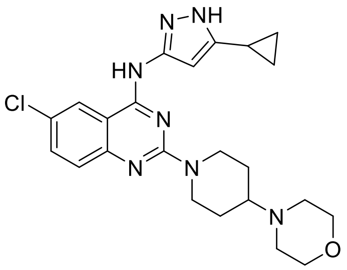

Compound **6b**

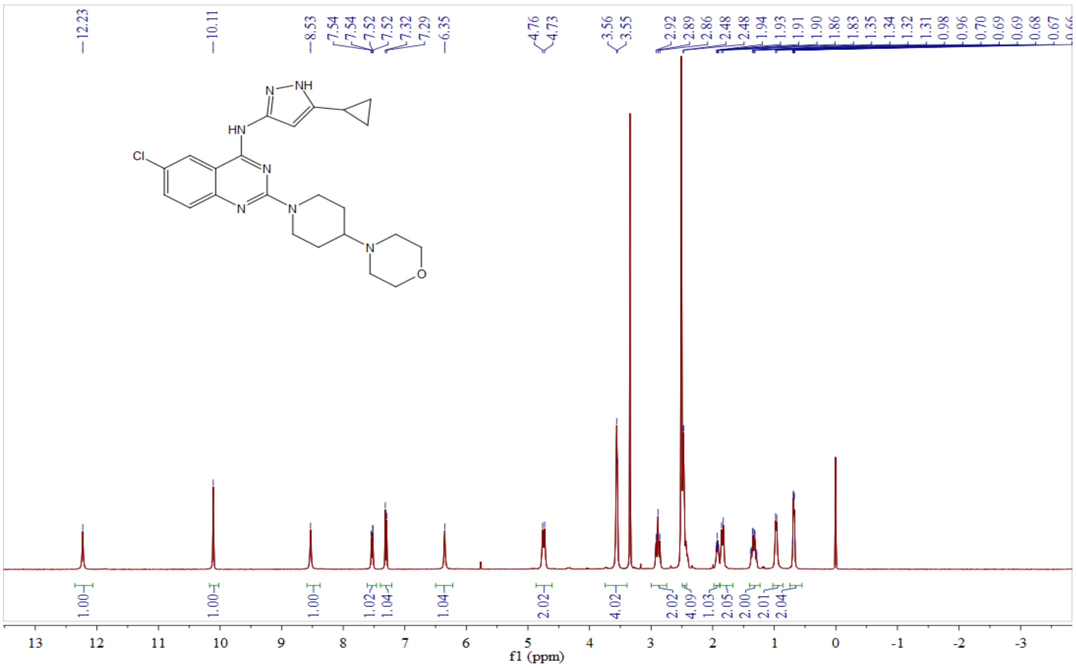

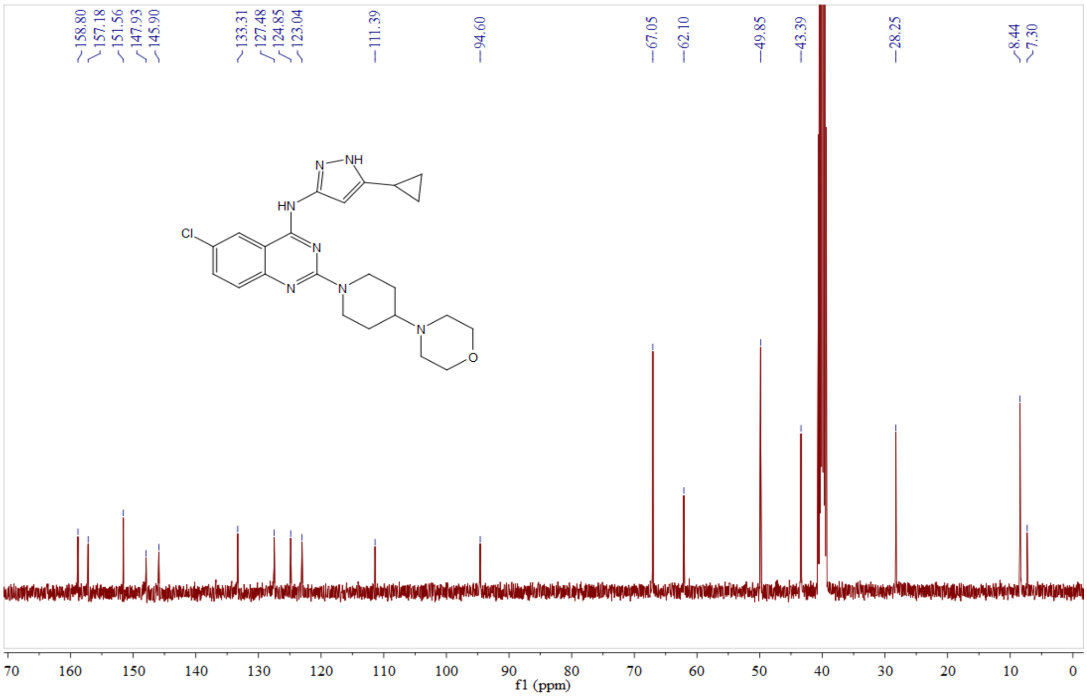

User Spectra

Fragmentor Voltage	Collision Energy	Ionization Mode
200	0	ESI

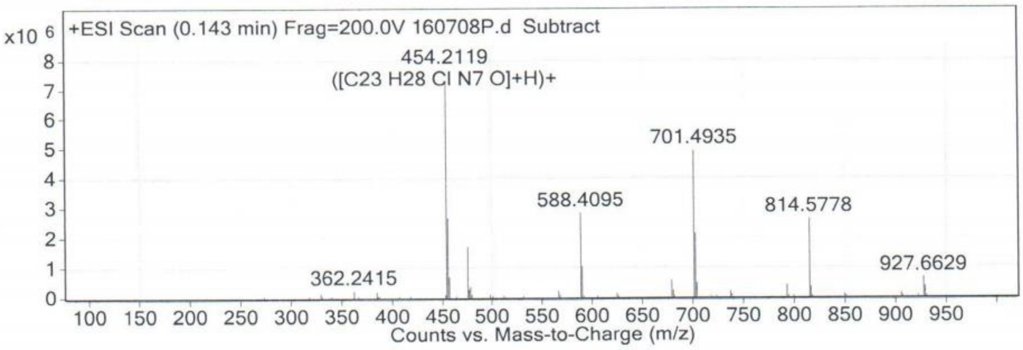

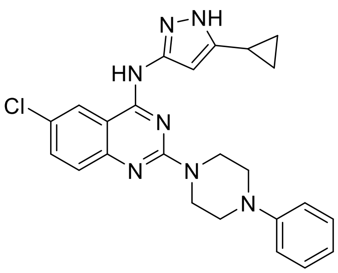

Compound **7a**

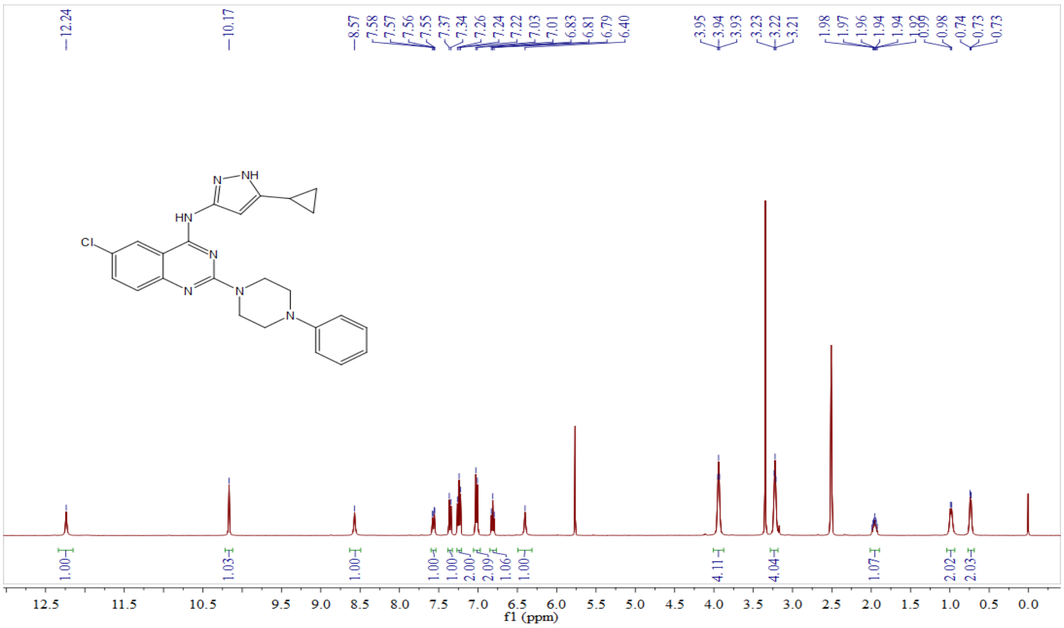
12.24
10.17
8.57 7.58 7.57 7.56 7.55 7.37 7.34 7.26 7.24 7.22 7.03 7.01 6.83 6.81 6.79 6.40
3.95 3.94 3.93 3.23 3.22 3.21
1.98 1.97 1.96 1.94 1.94 1.92 0.99 0.98 0.74 0.73 0.73
1.00 1.03 1.00 1.00 1.00 2.00 2.09 1.06 1.00 4.11 4.04 1.07 2.02 2.03
f1 (ppm)

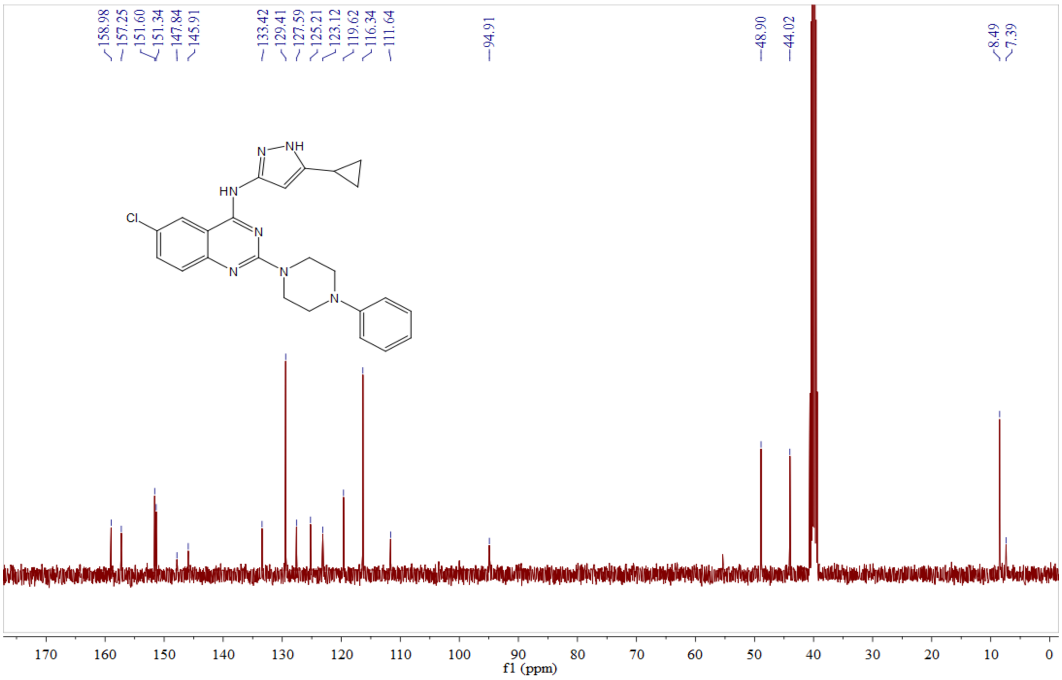
158.98 157.25 151.60 151.34 147.84 145.91
133.42 129.41 127.59 125.21 123.12 119.62 116.34 111.64
94.91
48.90 44.02
8.49 7.39
f1 (ppm)

User Spectra

Fragmentor Voltage	Collision Energy	Ionization Mode
200	0	ESI

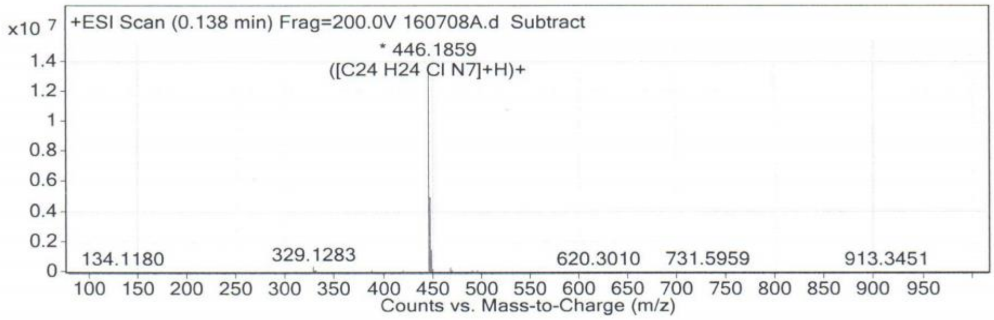

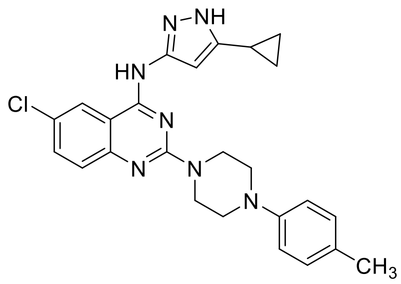
N
NH
HN
Cl
N
N
N
N
CH3

Compound **7b**

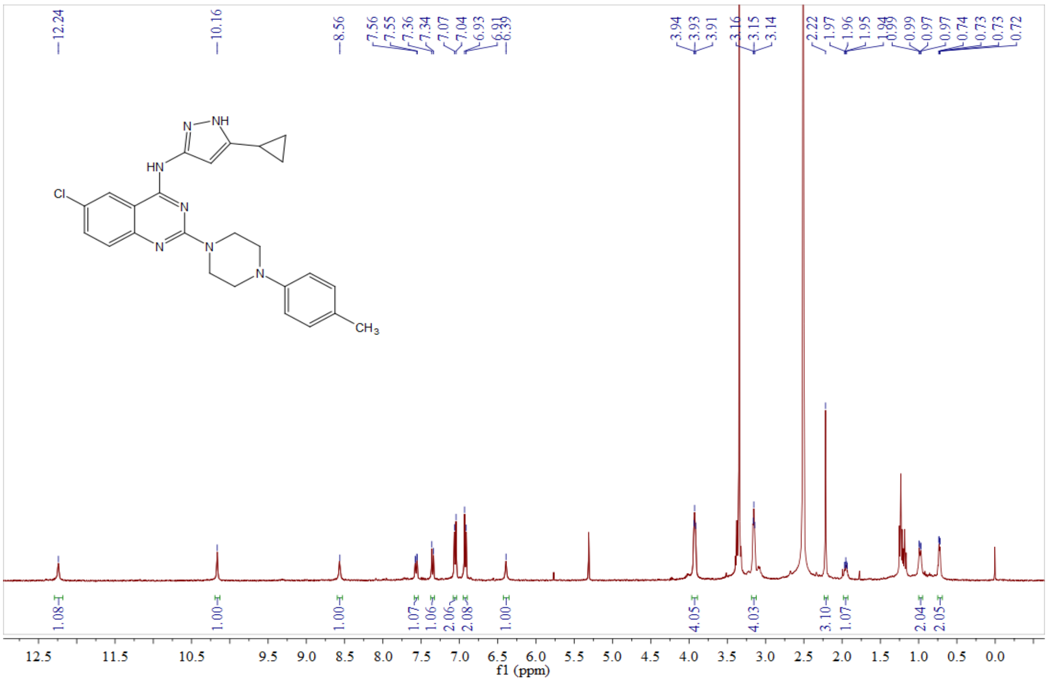
12.24
10.16
8.56
7.56
7.55
7.36
7.34
7.07
7.04
6.93
6.91
6.39
3.94
3.93
3.91
3.16
3.15
3.14
2.22
1.97
1.96
1.95
1.94
0.99
0.97
0.97
0.74
0.73
0.73
0.72
1.08
1.00
1.00
1.07
1.06
2.06
2.08
1.00
4.05
4.03
3.10
1.07
2.04
2.05
f1 (ppm)

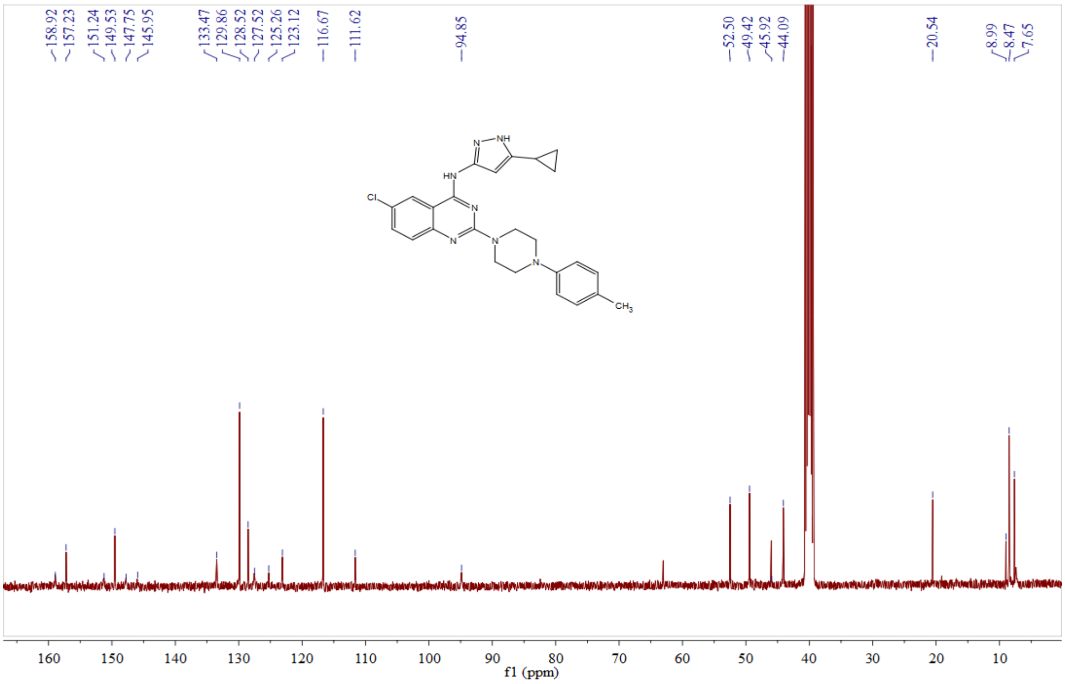
158.92
157.23
151.24
149.53
147.75
145.95
133.47
129.86
128.52
127.52
125.26
123.12
116.67
111.62
94.85
52.50
49.42
45.92
44.09
20.54
8.99
8.47
7.65
f1 (ppm)

User Spectra

Fragmentor Voltage	Collision Energy	Ionization Mode
200	0	ESI

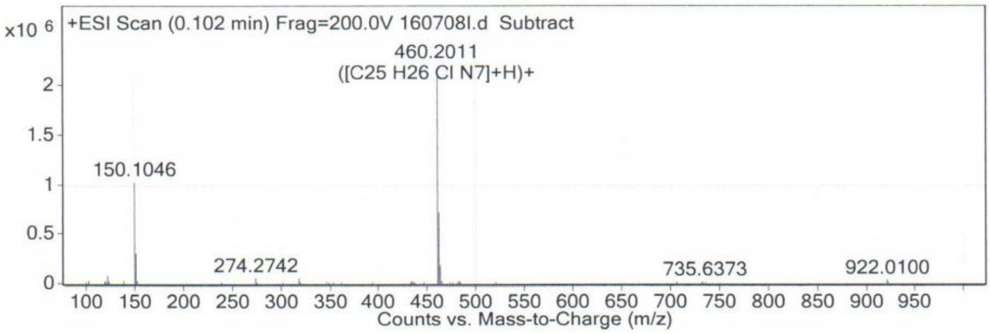

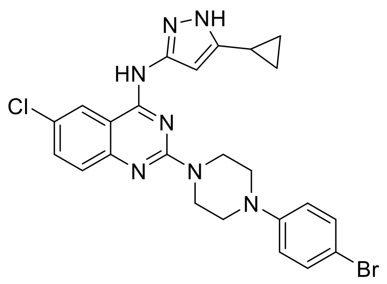

N
NH
HN
Cl
N
N
N
N
Br

Compound **7c**

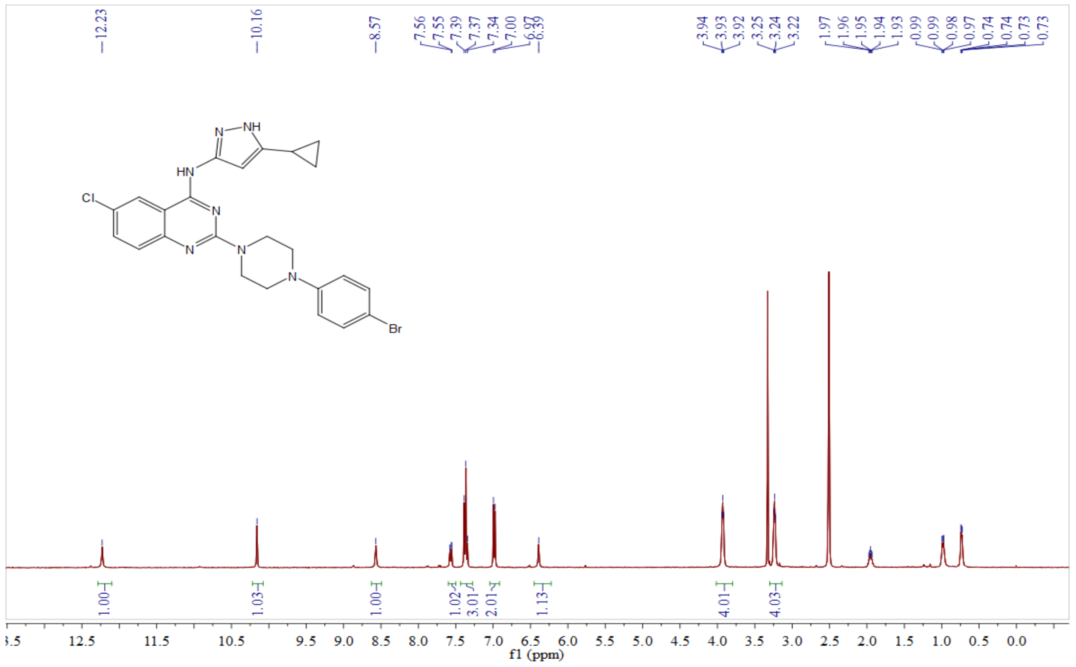

12.23
10.16
8.57
7.56
7.55
7.39
7.37
7.34
7.00
6.97
6.39
3.94
3.93
3.92
3.25
3.24
3.22
1.97
1.96
1.95
1.94
1.93
0.99
0.99
0.98
0.97
0.74
0.74
0.73
0.73
1.00
1.03
1.00
1.02
3.01
2.01
1.13
4.01
4.03
f1 (ppm)

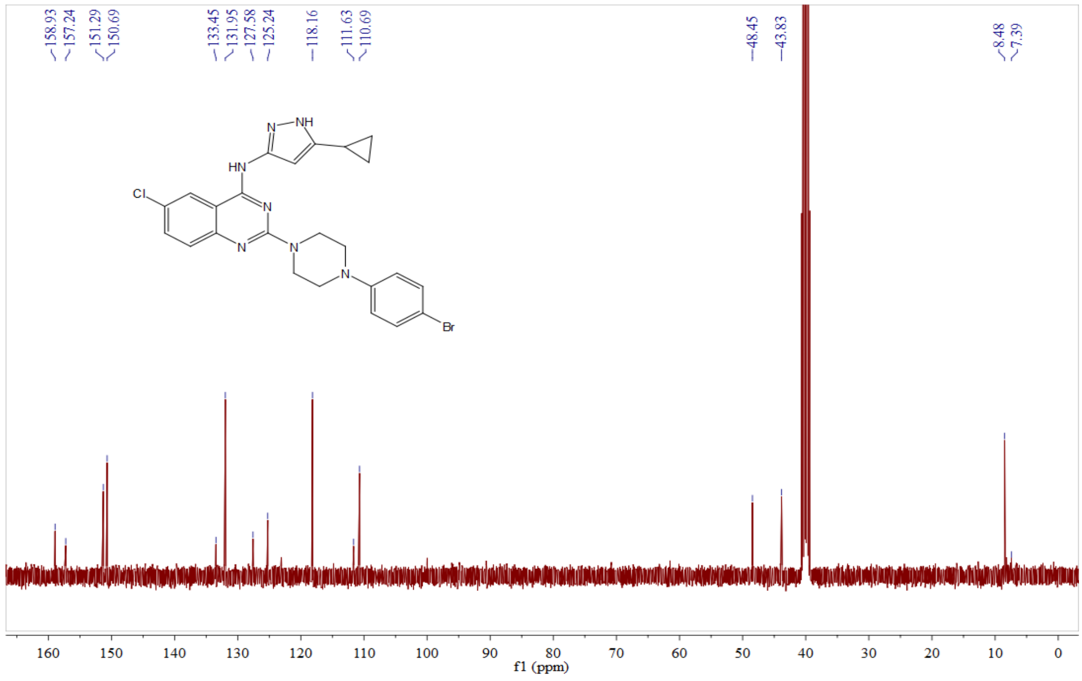

158.93
157.24
151.29
150.69
133.45
131.95
127.58
125.24
118.16
111.63
110.69
48.45
43.83
8.48
7.39
f1 (ppm)

User Spectra

Fragmentor Voltage	Collision Energy	Ionization Mode
200	0	ESI

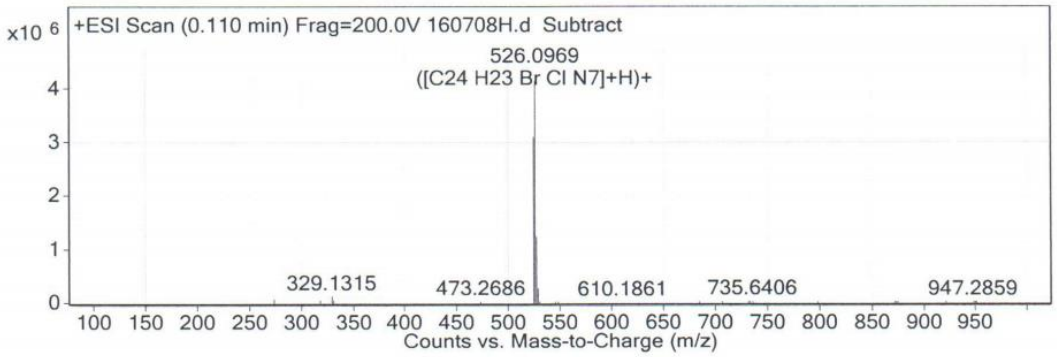

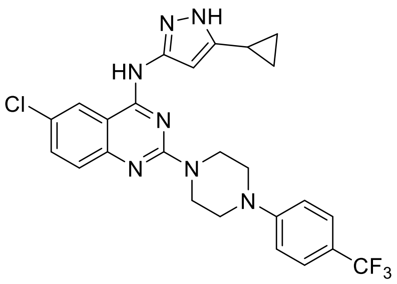

Compound **7d**

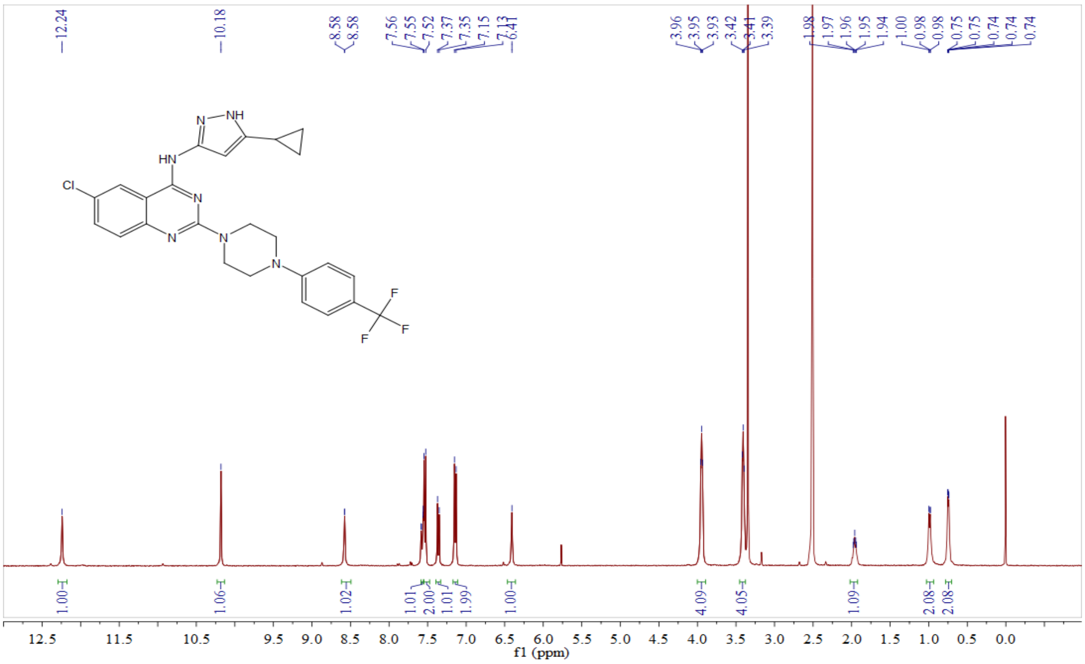

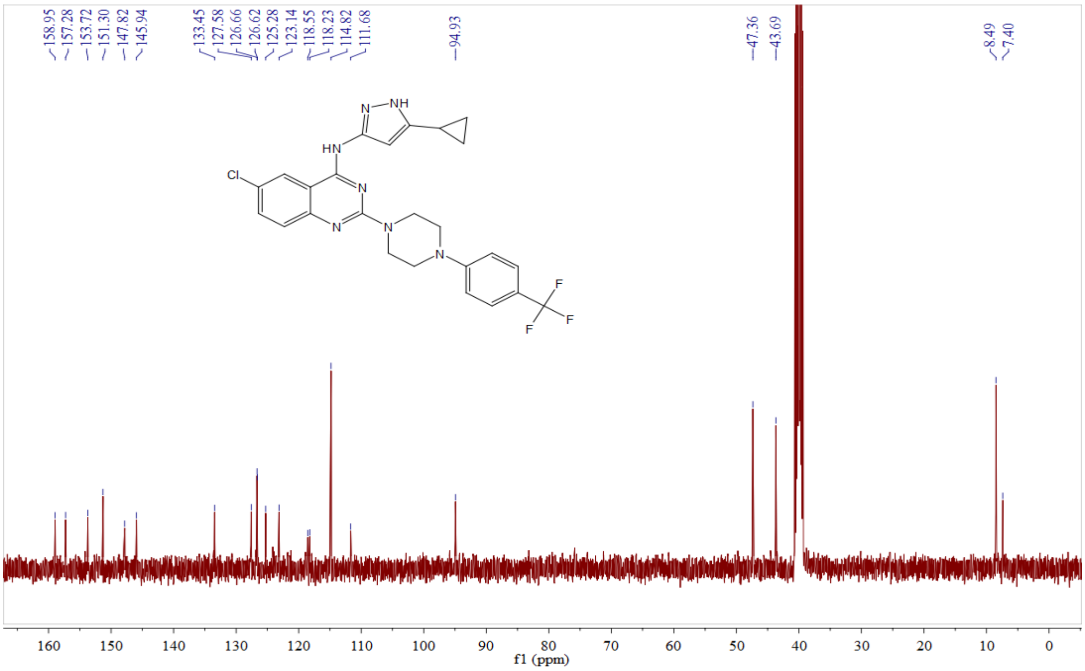

User Spectra

Fragmentor Voltage	Collision Energy	Ionization Mode
200	0	ESI

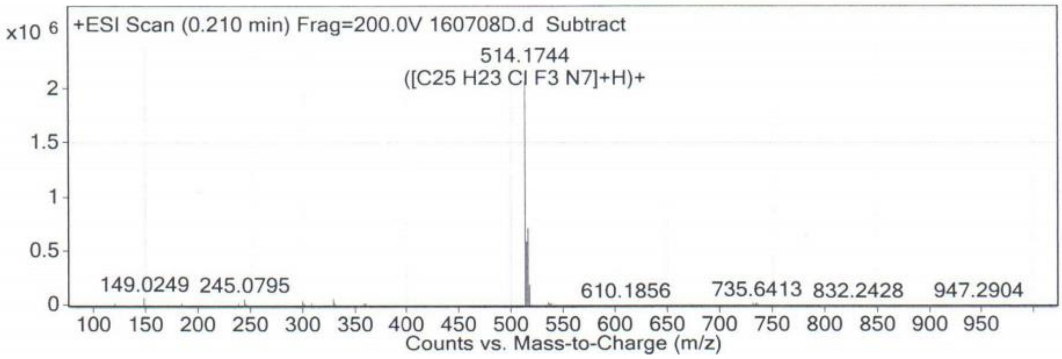

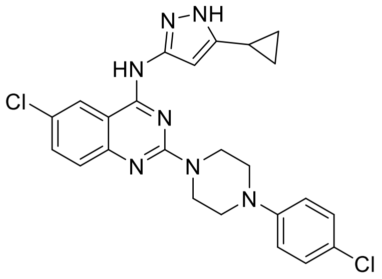

N
NH
HN
Cl
N
N
N
N
Cl

Compound **7e**

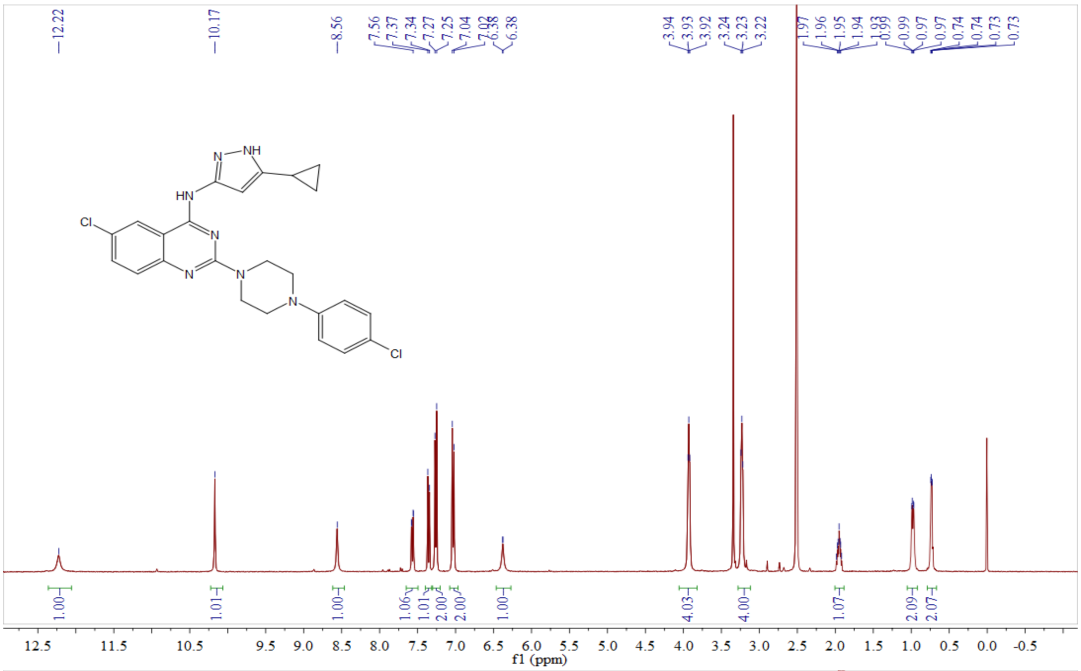

12.22
10.17
8.56
7.56
7.37
7.34
7.27
7.25
7.04
7.02
6.38
6.38
3.94
3.93
3.92
3.24
3.23
3.22
1.97
1.96
1.95
1.94
1.93
0.99
0.99
0.97
0.97
0.74
0.74
0.73
0.73
1.00
1.01
1.00
1.06
1.01
2.00
2.00
1.00
4.03
4.00
1.07
2.09
2.07
f1 (ppm)

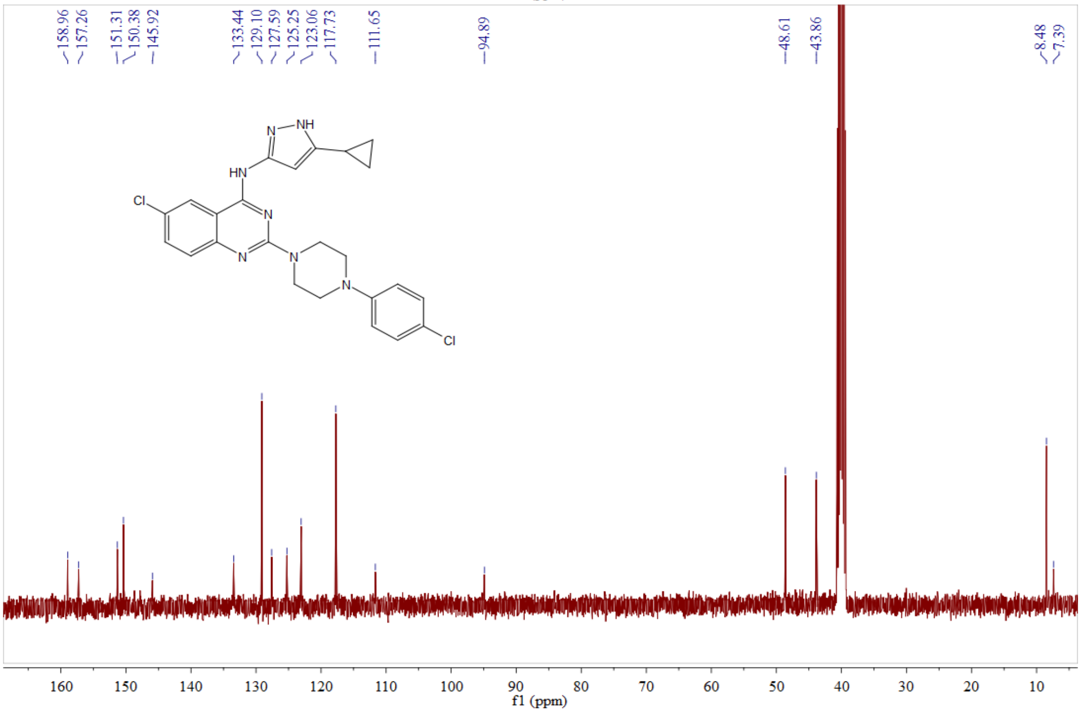

158.96
157.26
151.31
150.38
145.92
133.44
129.10
127.59
125.25
123.06
117.73
111.65
94.89
48.61
43.86
8.48
7.39
f1 (ppm)

User Spectra

Fragmentor Voltage	Collision Energy	Ionization Mode
200	0	ESI

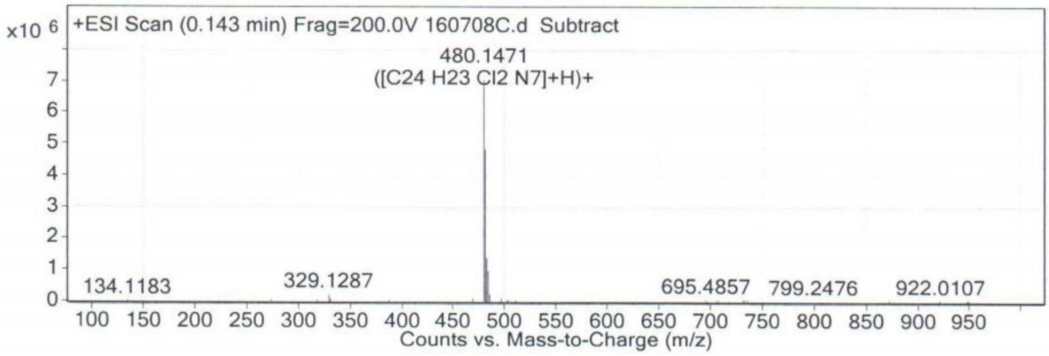

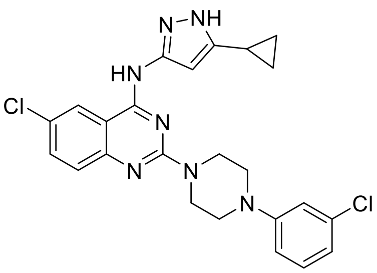
N
NH
HN
Cl
N
N
N
N
Cl

Compound **7f**

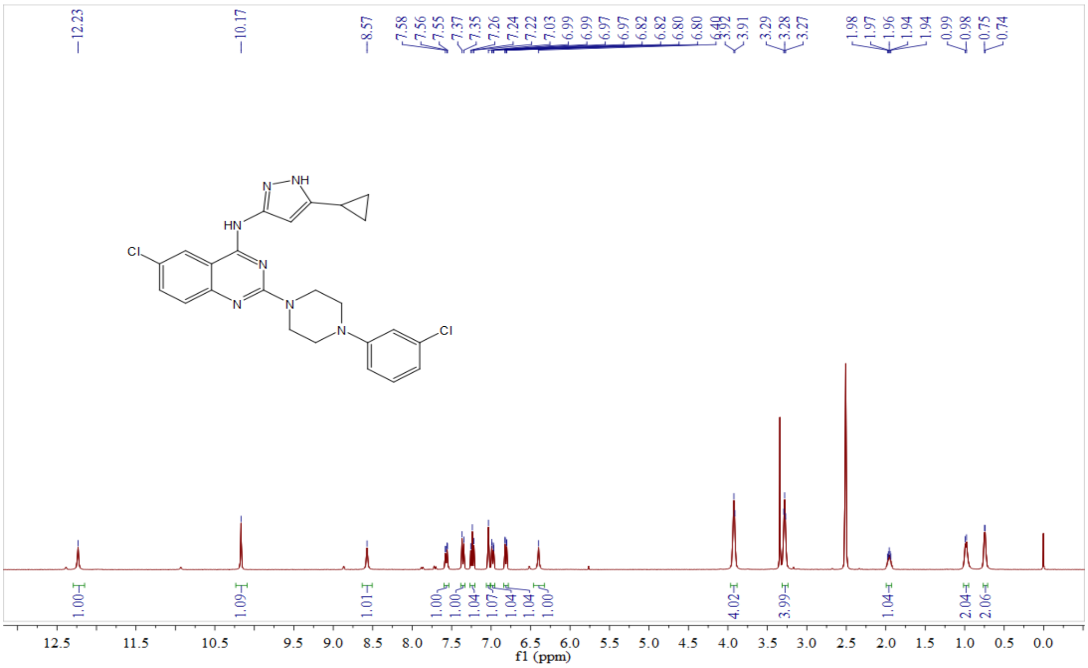
12.23
10.17
8.57
12.5
11.5
10.5
9.5
9.0
8.5
8.0
7.5
7.0
6.5
6.0
5.5
5.0
4.5
4.0
3.5
3.0
2.5
2.0
1.5
1.0
0.5
0.0
f1 (ppm)

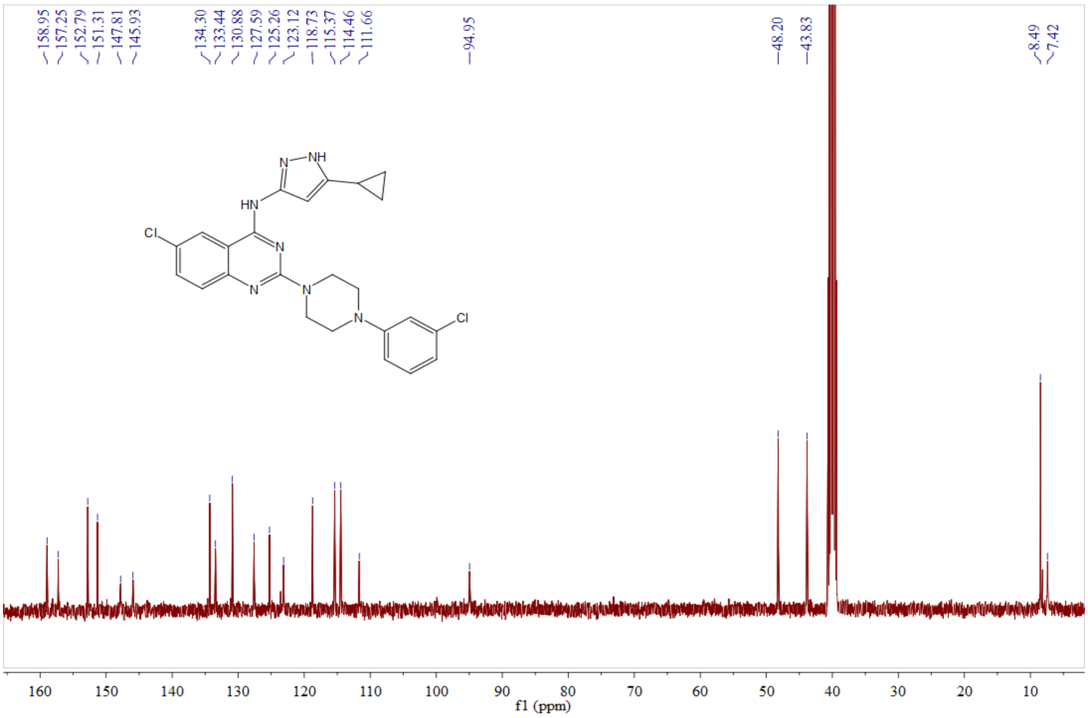
94.95
48.20
43.83
160
150
140
130
120
110
100
90
80
70
60
50
40
30
20
10
f1 (ppm)

User Spectra

Fragmentor Voltage	Collision Energy	Ionization Mode
200	0	ESI

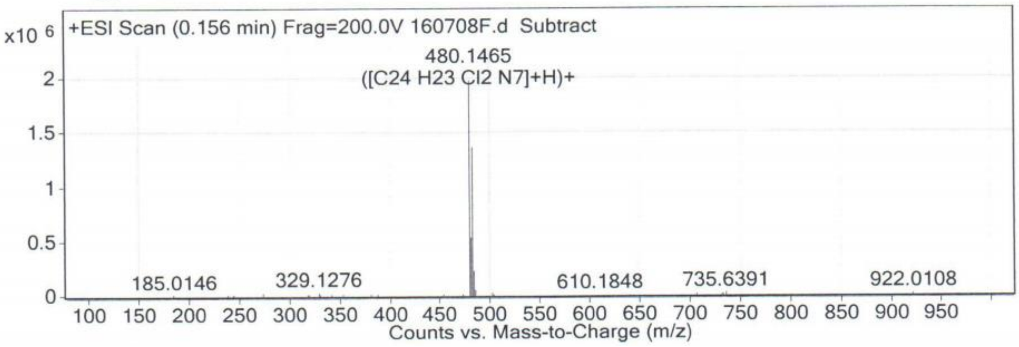

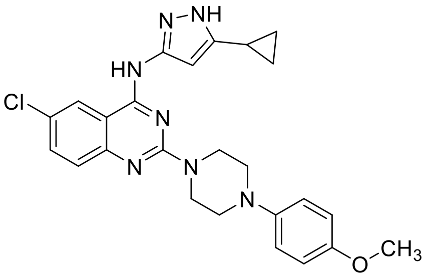

Compound **7g**

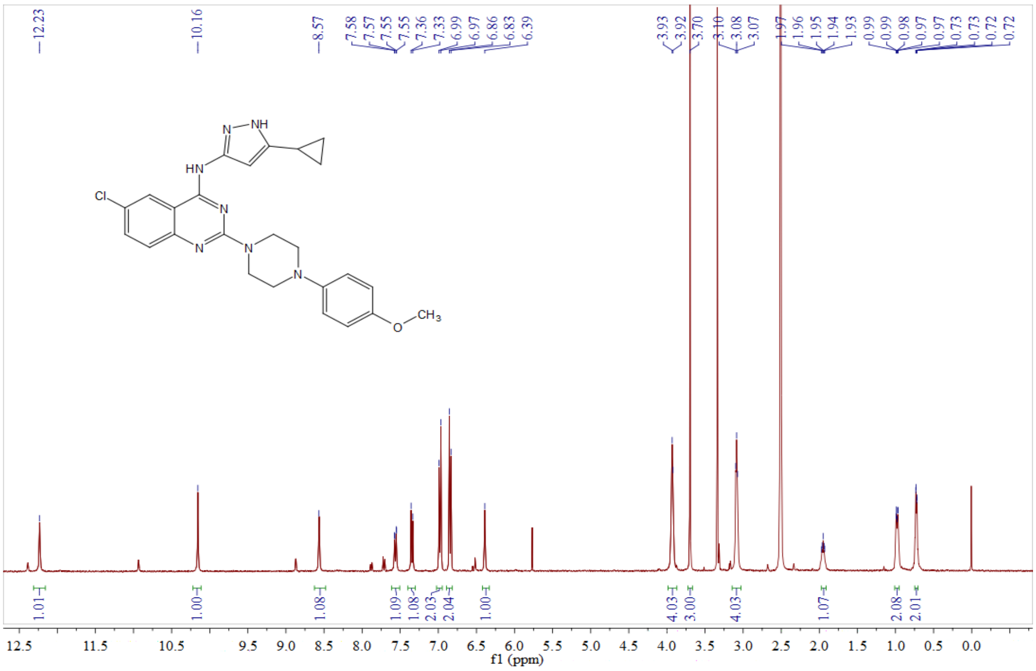

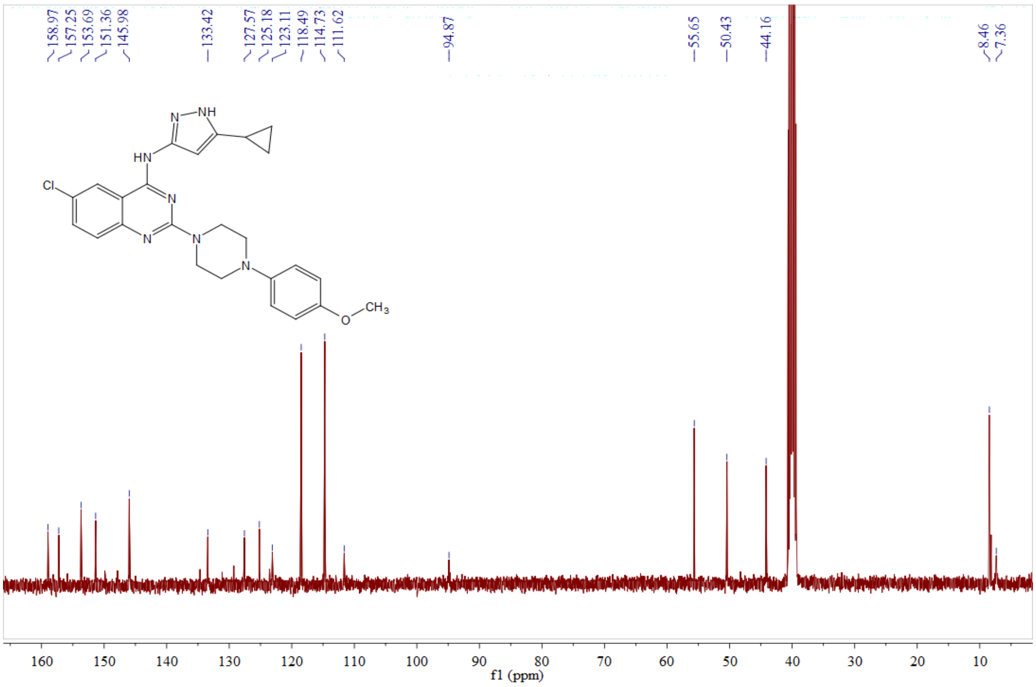

User Spectra

Fragmentor Voltage	Collision Energy	Ionization Mode
200	0	ESI

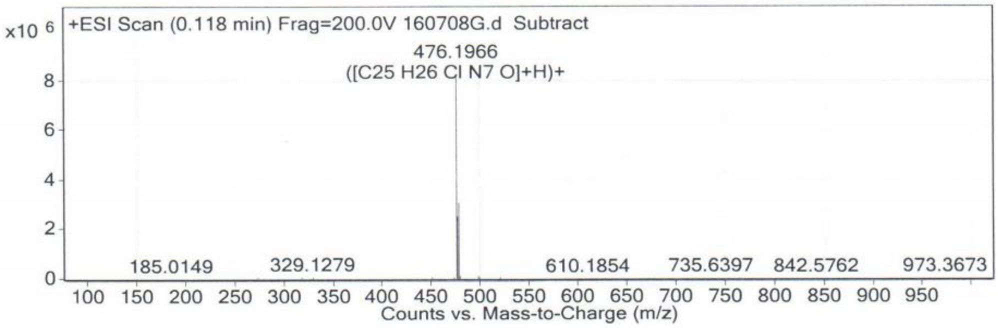

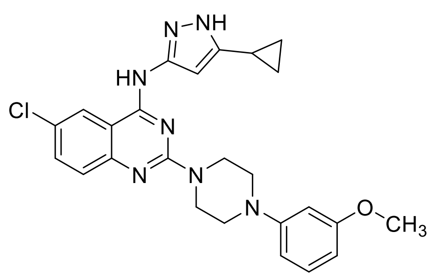

Compound **7h**

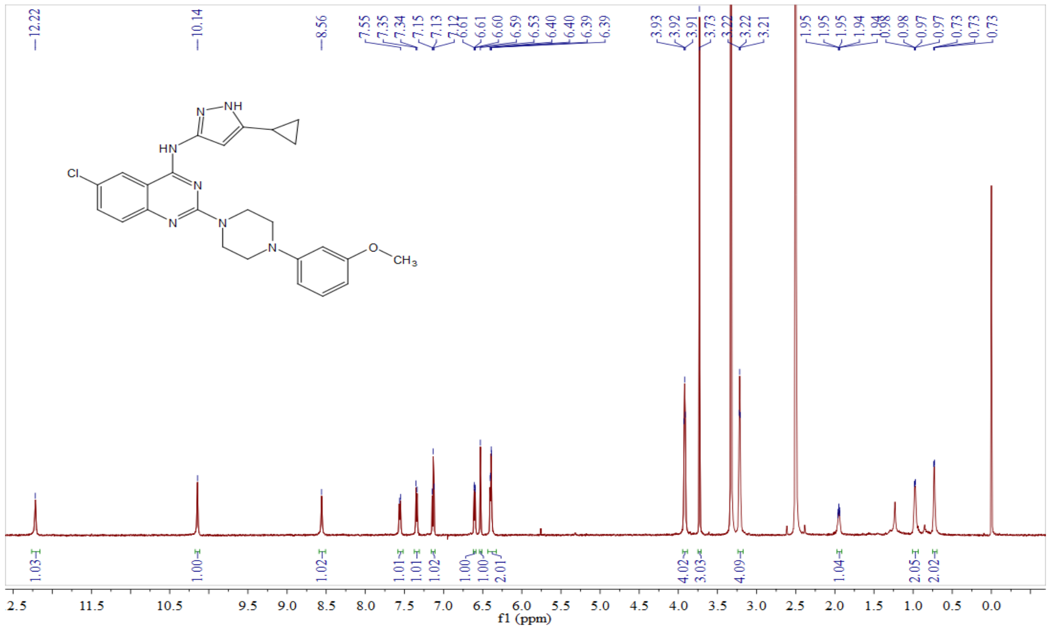

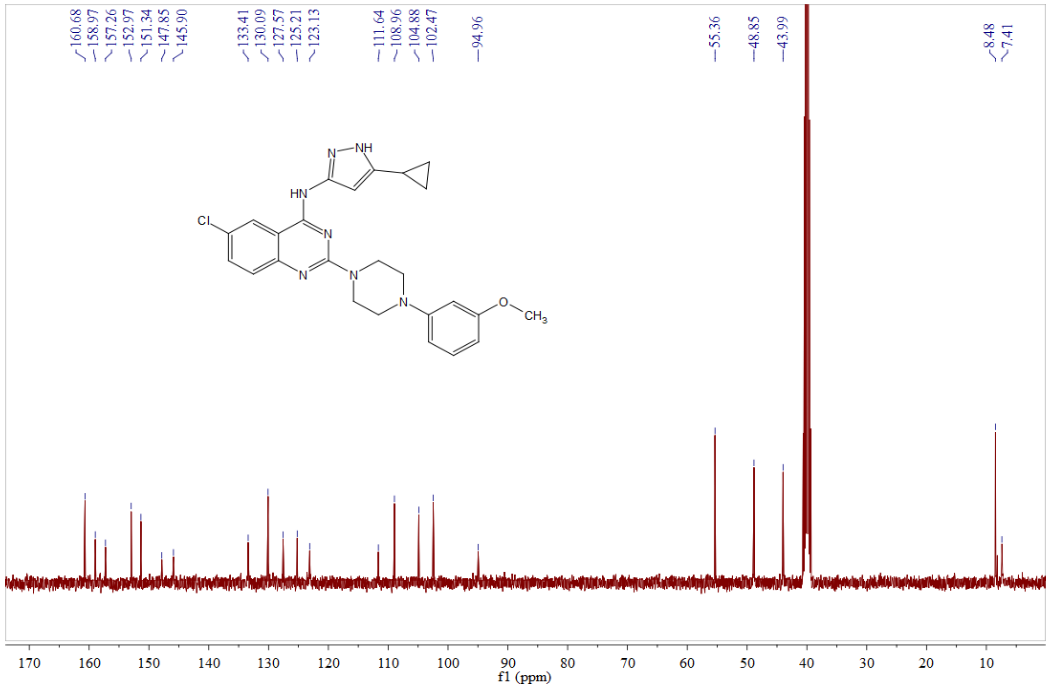

User Spectra

Fragmentor Voltage	Collision Energy	Ionization Mode
200	0	ESI

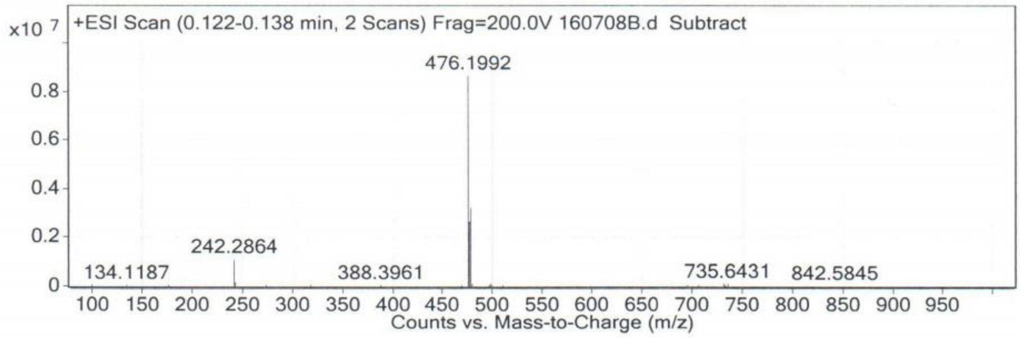

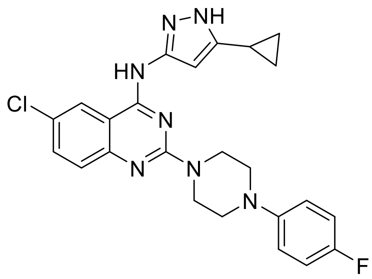
N–NH
HN
Cl
N
N
N
N
F

Compound **7i**

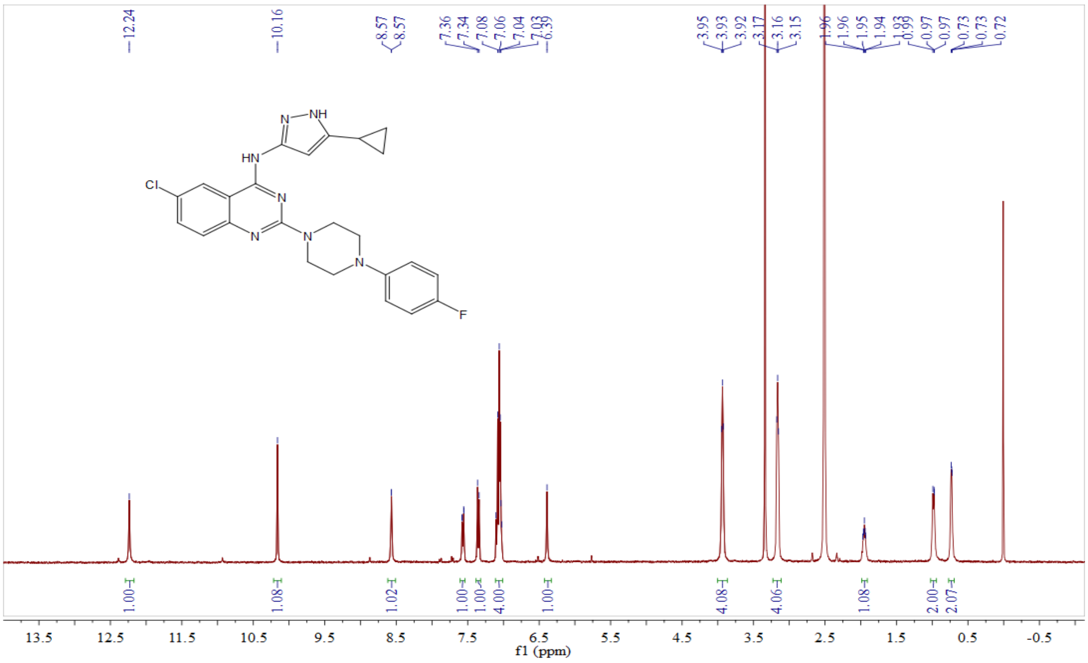
12.24
10.16
8.57
8.57
7.36
7.34
7.08
7.06
7.04
7.03
6.39
3.95
3.93
3.92
3.17
3.16
3.15
1.96
1.96
1.95
1.94
1.93
0.99
0.97
0.97
0.73
0.73
0.72
1.00
1.08
1.02
1.00
1.00
4.00
1.00
4.08
4.06
1.08
2.00
2.07
13.5
12.5
11.5
10.5
9.5
8.5
7.5
6.5
5.5
4.5
3.5
2.5
1.5
0.5
-0.5
f1 (ppm)

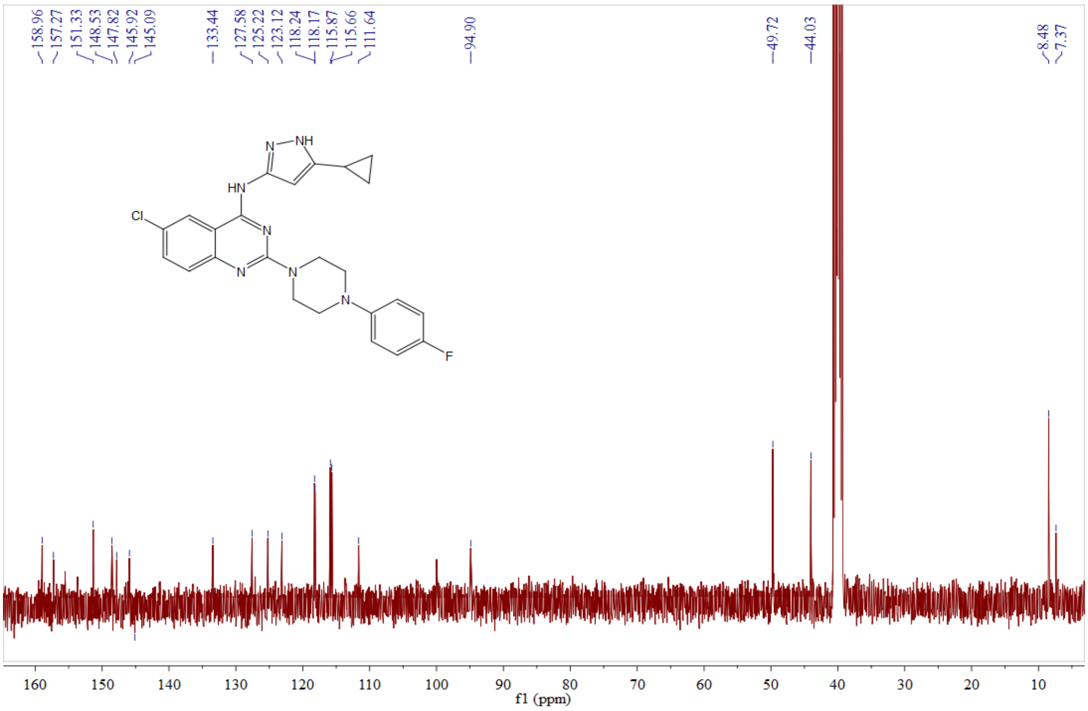
158.96
157.27
151.33
148.53
147.82
145.92
145.09
133.44
127.58
125.22
123.12
118.24
118.17
115.87
115.66
111.64
94.90
49.72
44.03
8.48
7.37
160
150
140
130
120
110
100
90
80
70
60
50
40
30
20
10
f1 (ppm)

User Spectra

Fragmentor Voltage	Collision Energy	Ionization Mode
200	0	ESI

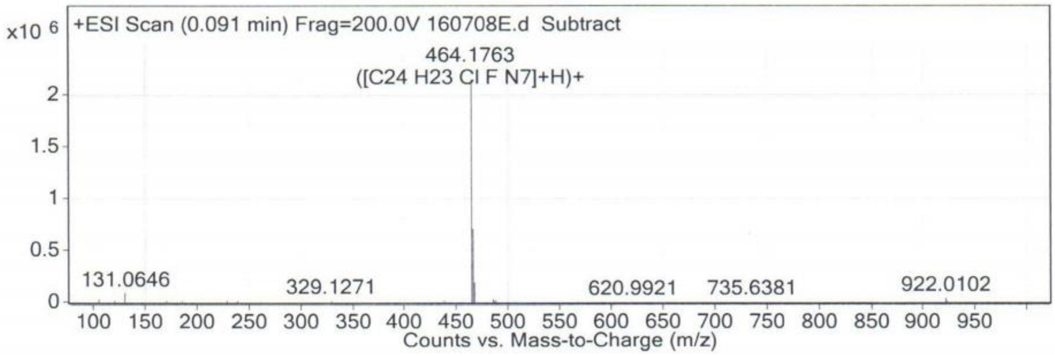

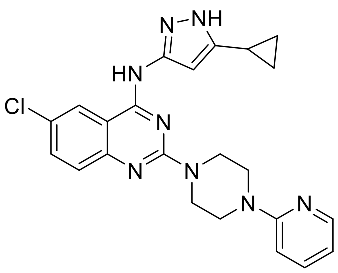

Compound **8a**

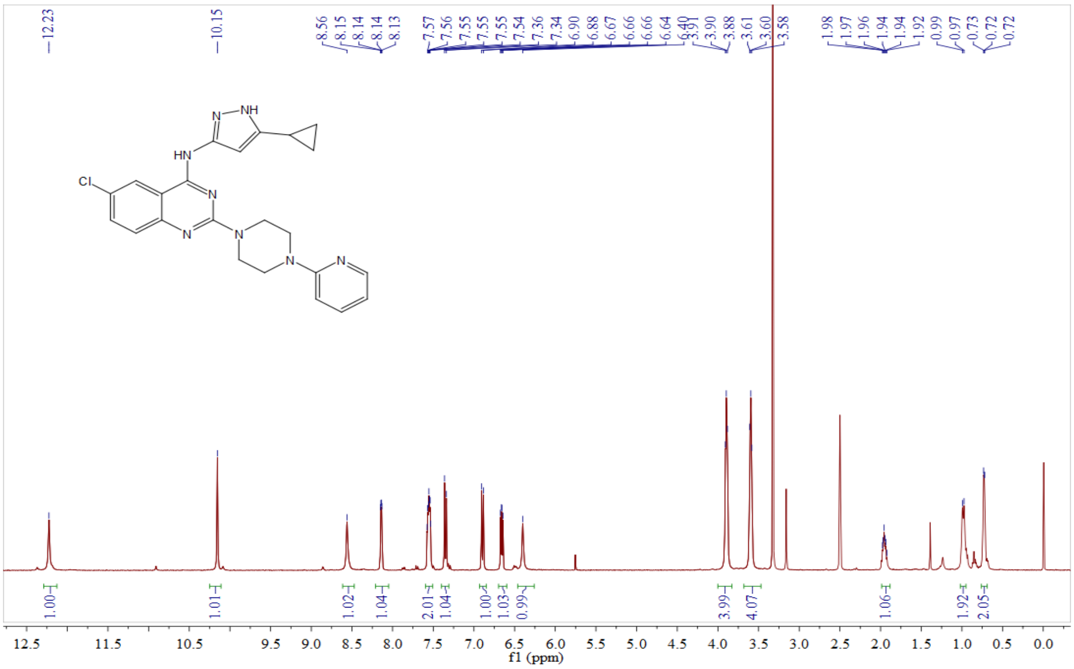

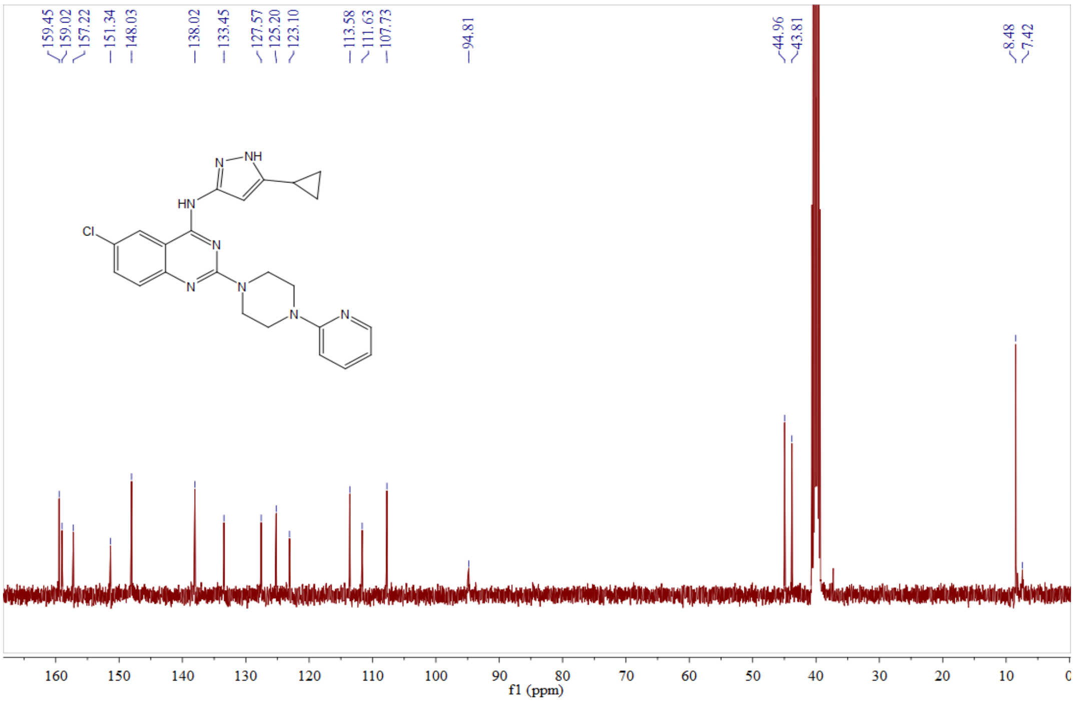

User Spectra

Fragmentor Voltage	Collision Energy	Ionization Mode
200	0	ESI

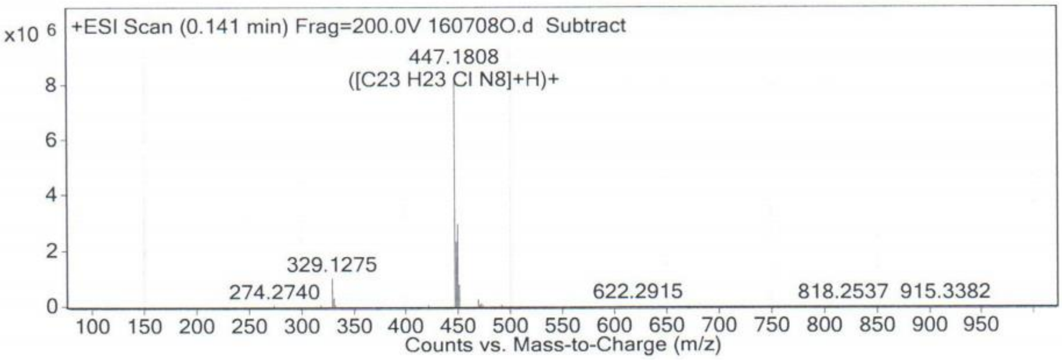

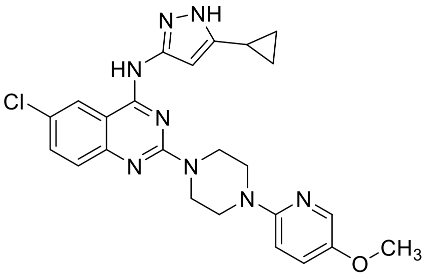

Compound **8b**

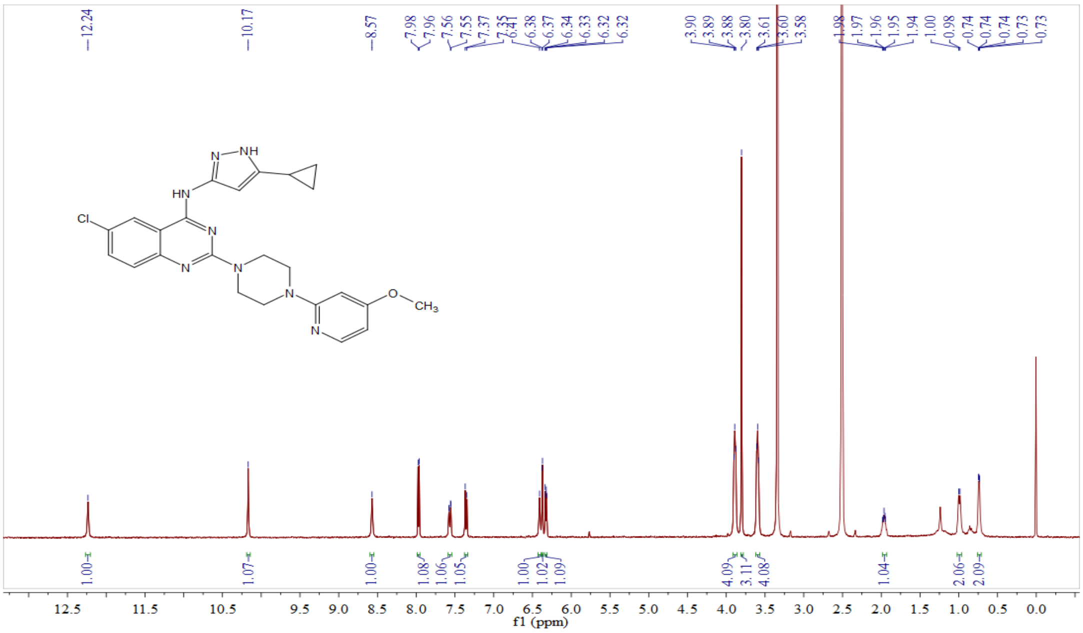

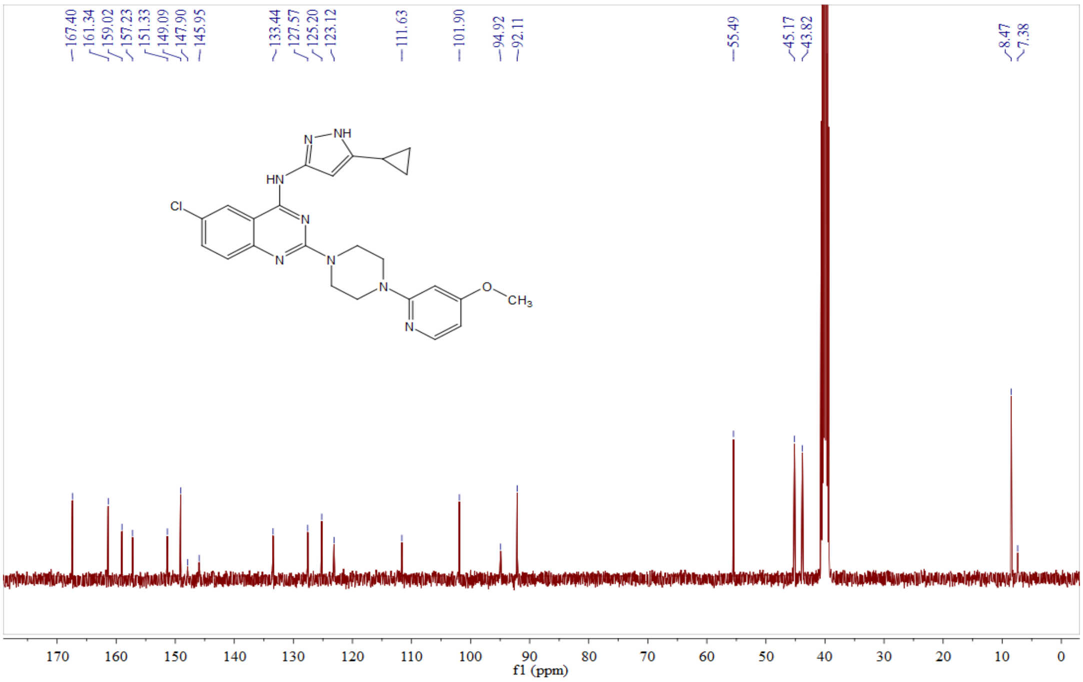

User Spectra

Fragmentor Voltage	Collision Energy	Ionization Mode
200	0	ESI

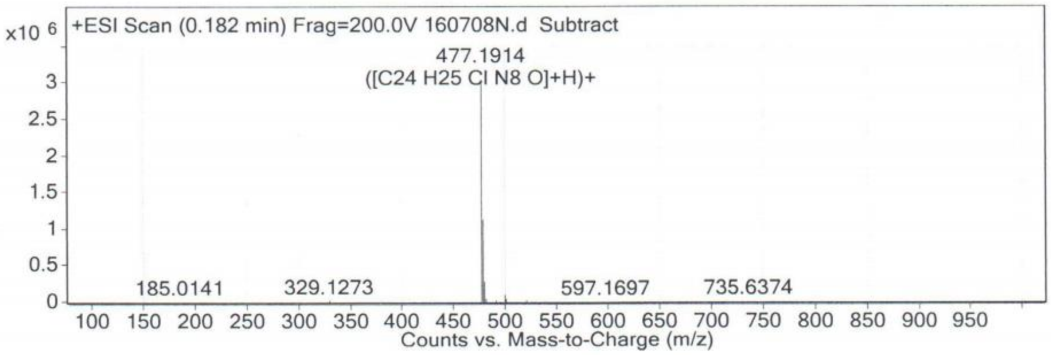

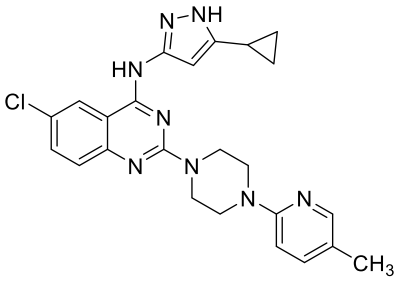
N
NH
HN
Cl
N
N
N
N
N
CH3

Compound **8c**

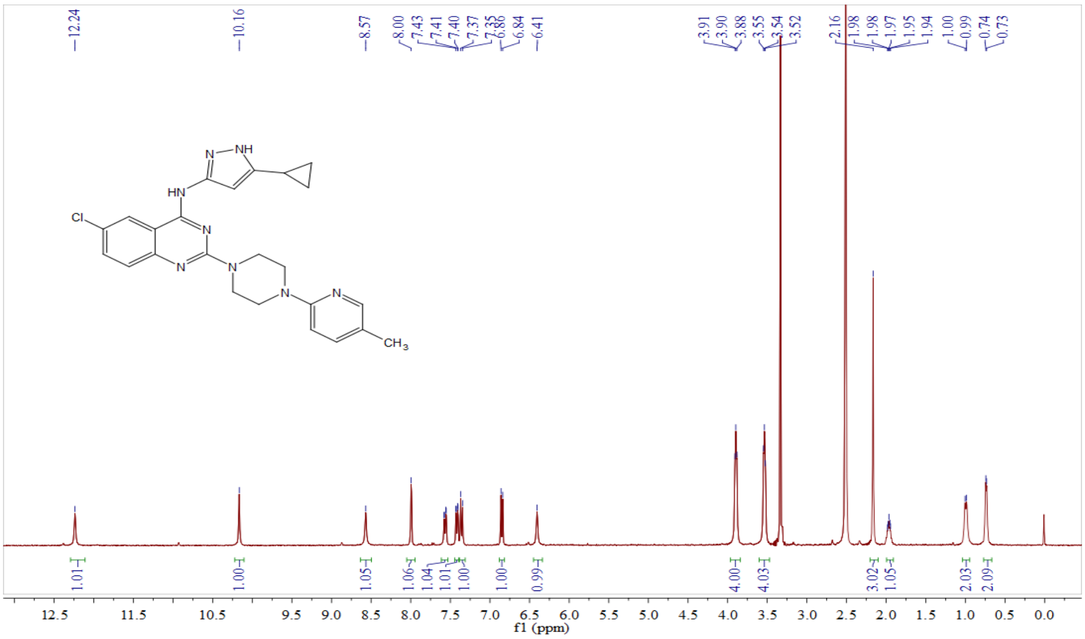
12.24
10.16
8.57
8.00
7.43
7.41
7.40
7.37
7.35
6.86
6.84
6.41
3.91
3.90
3.88
3.55
3.54
3.52
2.16
1.98
1.98
1.97
1.95
1.94
1.00
0.99
0.74
0.73
1.01
1.00
1.05
1.06
1.04
1.01
1.00
1.00
0.99
4.00
4.03
3.02
1.05
2.03
2.09
f1 (ppm)

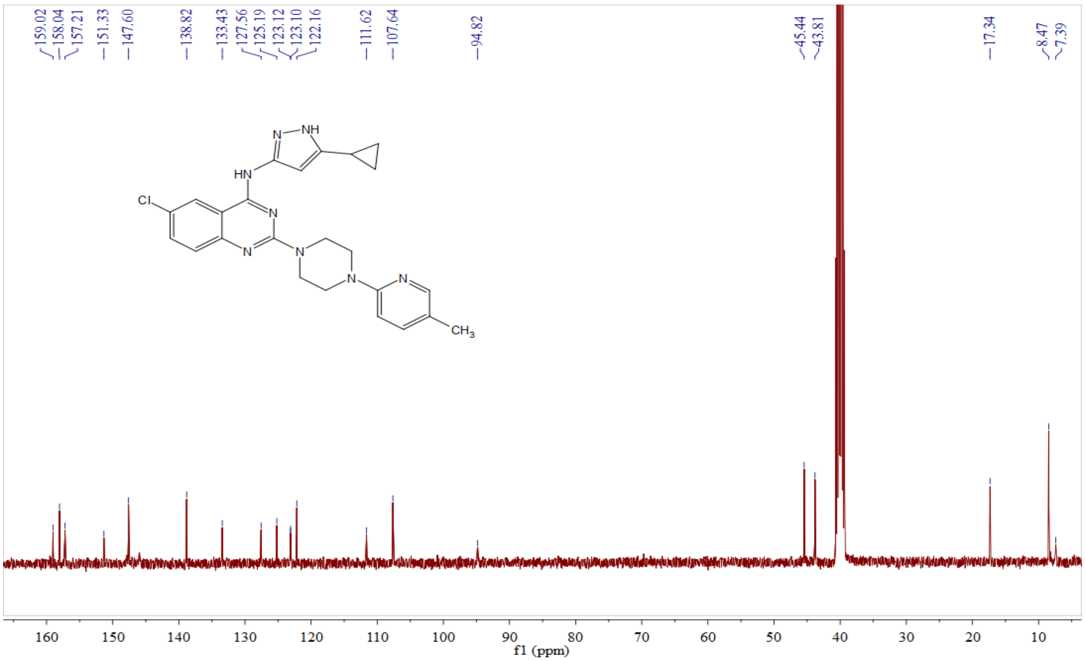
159.02
158.04
157.21
151.33
147.60
138.82
133.43
127.56
125.19
123.12
123.10
122.16
111.62
107.64
94.82
45.44
43.81
17.34
8.47
7.39
f1 (ppm)

User Spectra

Fragmentor Voltage	Collision Energy	Ionization Mode
200	0	ESI

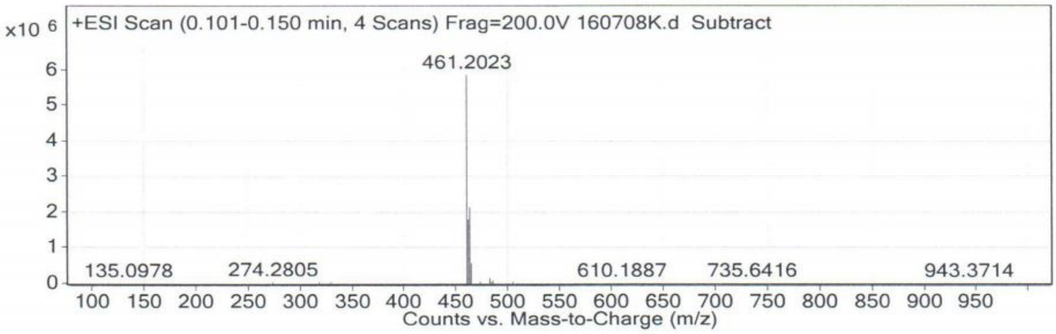

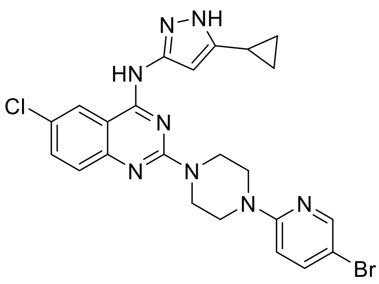

Compound **8d**

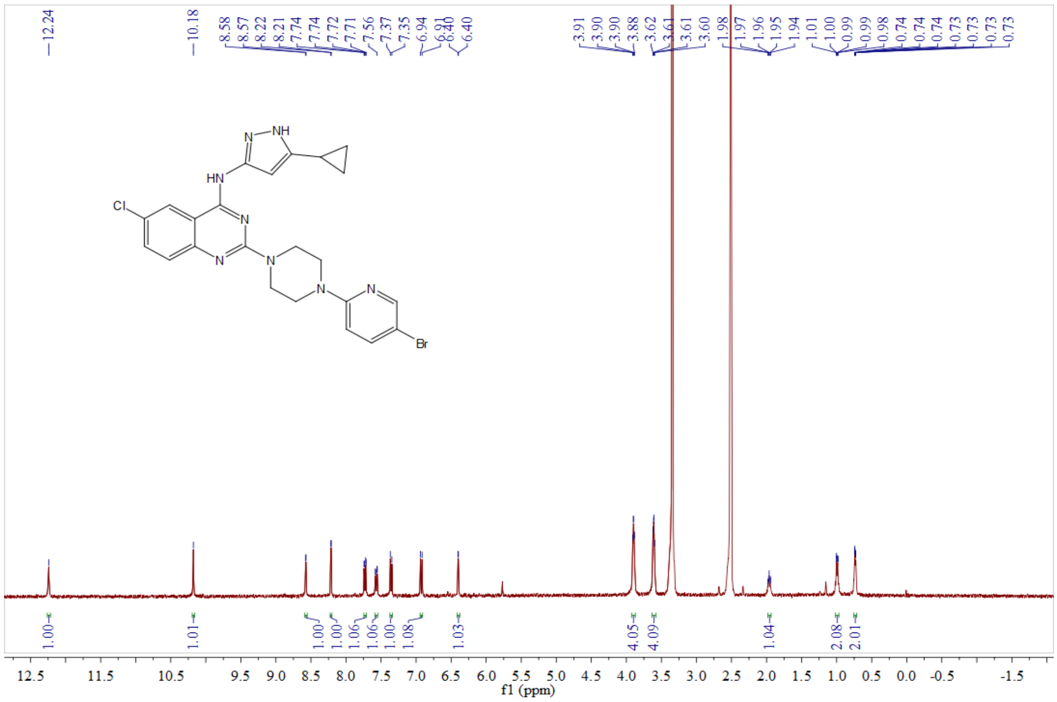

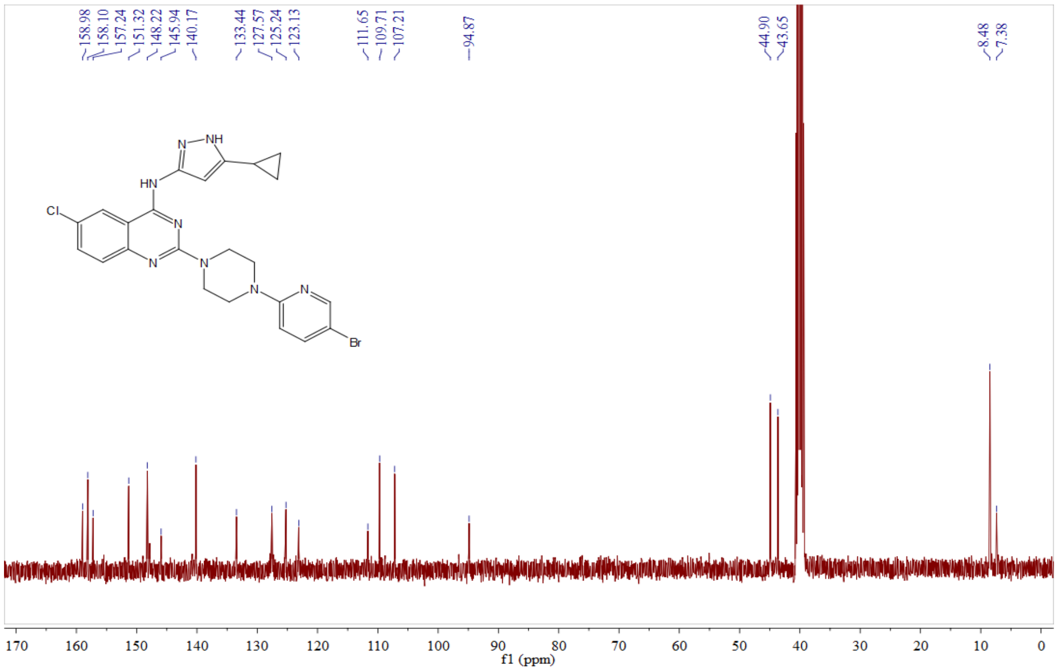

User Spectra

Fragmentor Voltage	Collision Energy	Ionization Mode
200	0	ESI

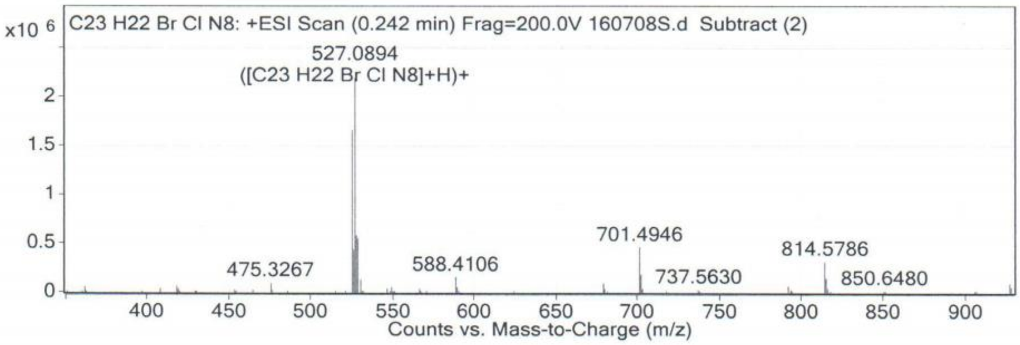

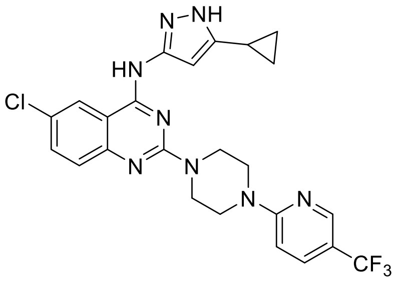

Compound **8e**

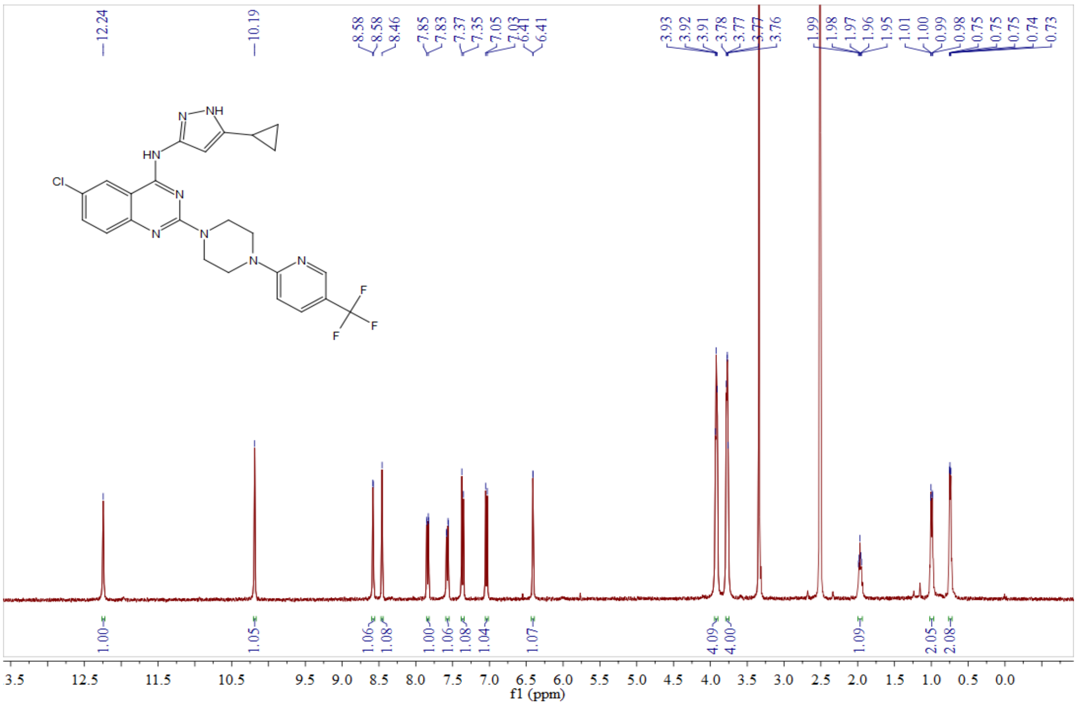
12.24
10.19
8.58
8.58
8.46
7.85
7.83
7.37
7.35
7.05
7.03
6.41
6.41
3.93
3.92
3.91
3.78
3.77
3.77
3.76
1.99
1.98
1.97
1.96
1.95
1.01
1.00
0.99
0.98
0.75
0.75
0.75
0.74
0.73
1.00
1.05
1.06
1.08
1.00
1.06
1.08
1.04
1.07
4.09
4.00
1.09
2.05
2.08
f1 (ppm)

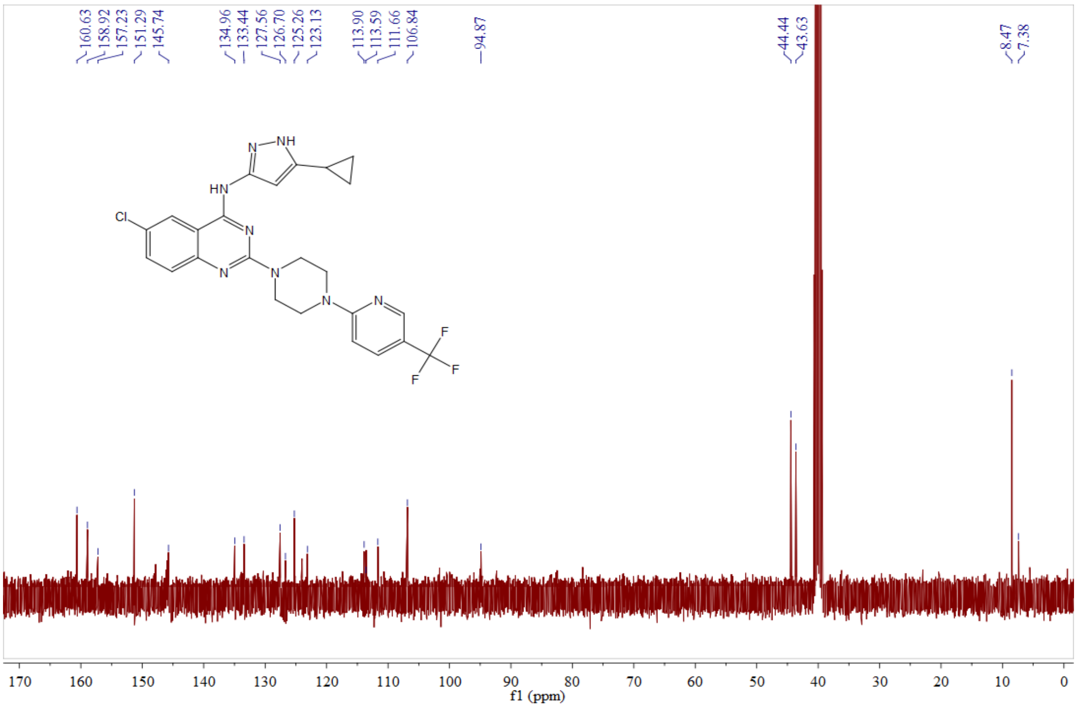
160.63
158.92
157.23
151.29
145.74
134.96
133.44
127.56
126.70
125.26
123.13
113.90
113.59
111.66
106.84
94.87
44.44
43.63
8.47
7.38
f1 (ppm)

User Spectra

Fragmentor Voltage	Collision Energy	Ionization Mode
200	0	ESI

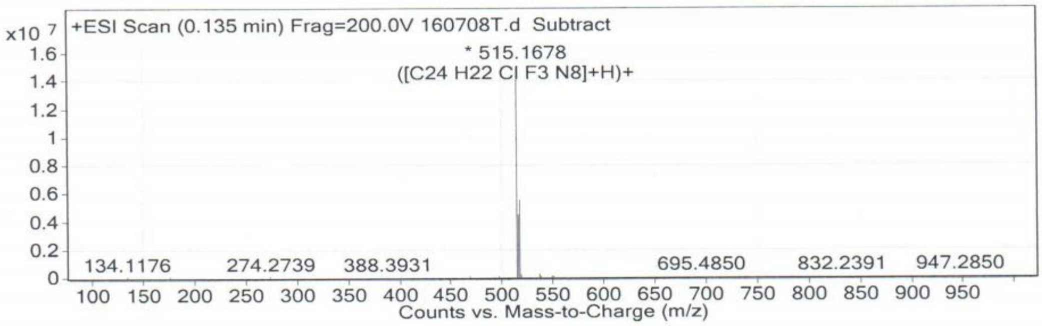

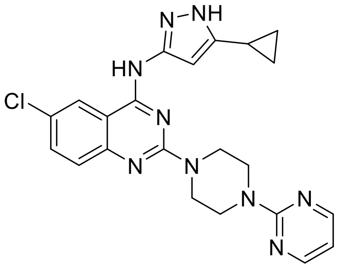
N
NH
HN
Cl
N
N
N
N
N
N

Compound **9a**

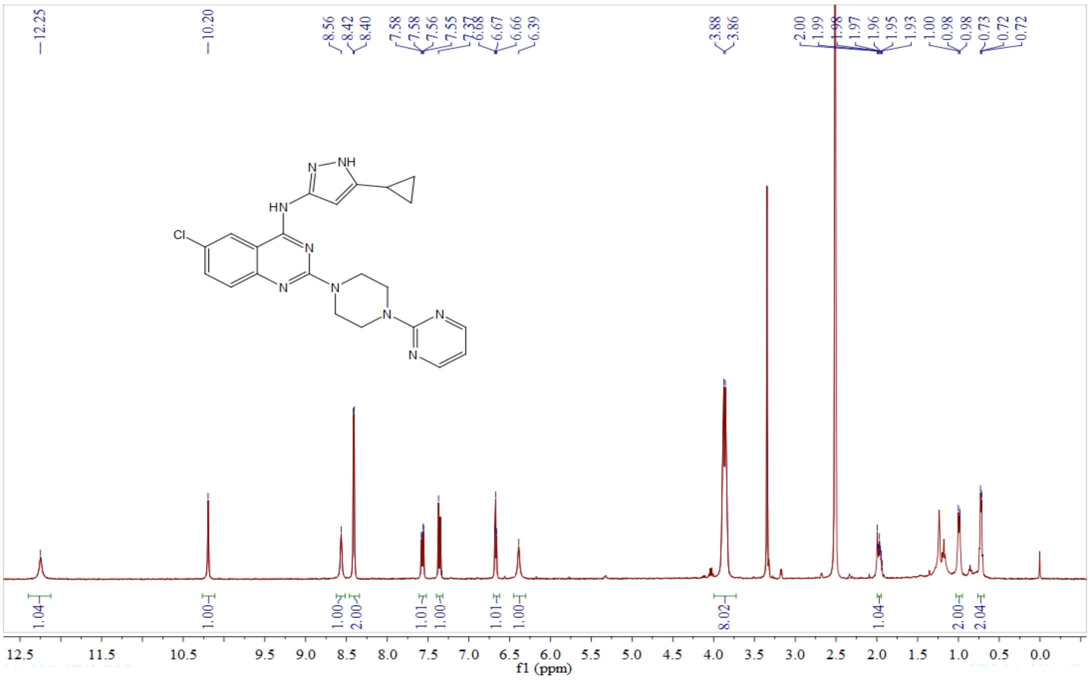
12.25
10.20
8.56
8.42
8.40
7.58
7.58
7.56
7.55
7.37
6.68
6.67
6.66
6.39
3.88
3.86
2.00
1.99
1.98
1.97
1.96
1.95
1.93
1.00
0.98
0.98
0.73
0.72
0.72
1.04
1.00
1.00
2.00
1.01
1.00
1.01
1.00
8.02
1.04
2.00
2.04
f1 (ppm)

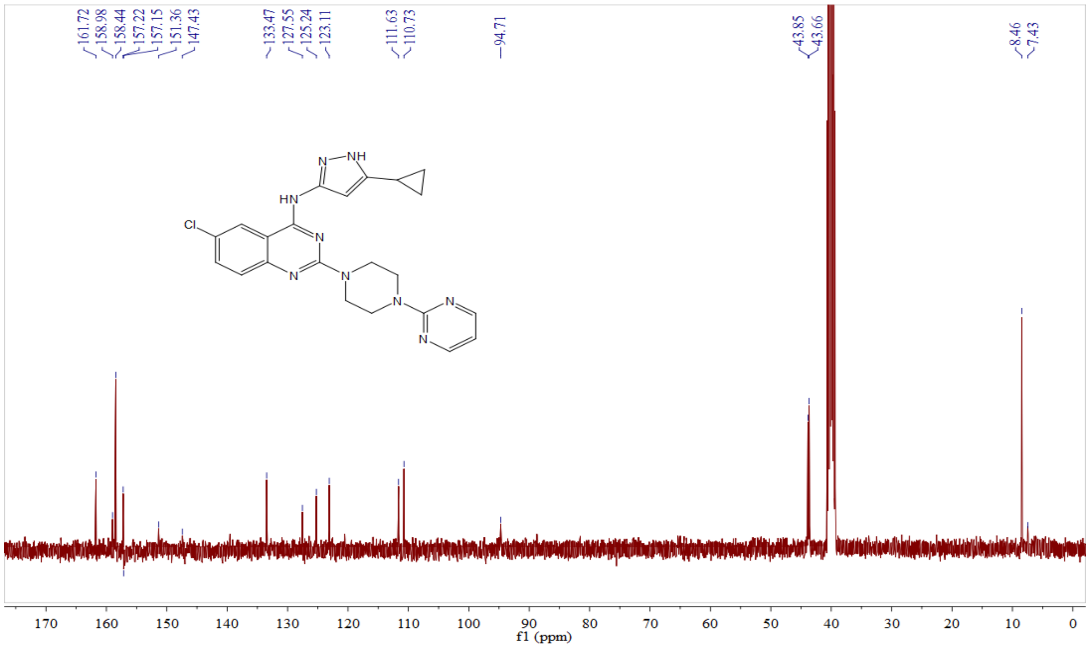
161.72
158.98
158.44
157.22
157.15
151.36
147.43
133.47
127.55
125.24
123.11
111.63
110.73
94.71
43.85
43.66
8.46
7.43
f1 (ppm)

User Spectra

Fragmentor Voltage	Collision Energy	Ionization Mode
200	0	ESI

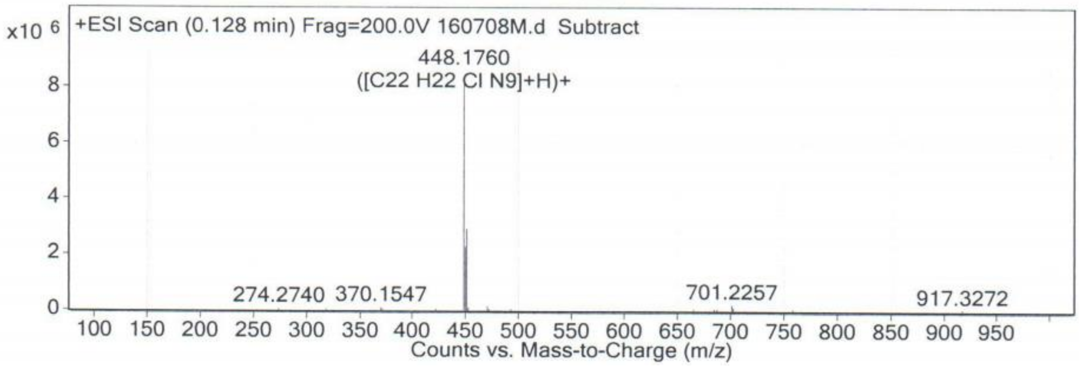

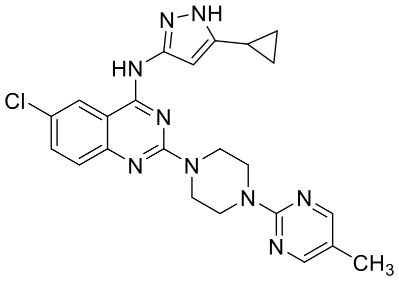

Compound **9b**

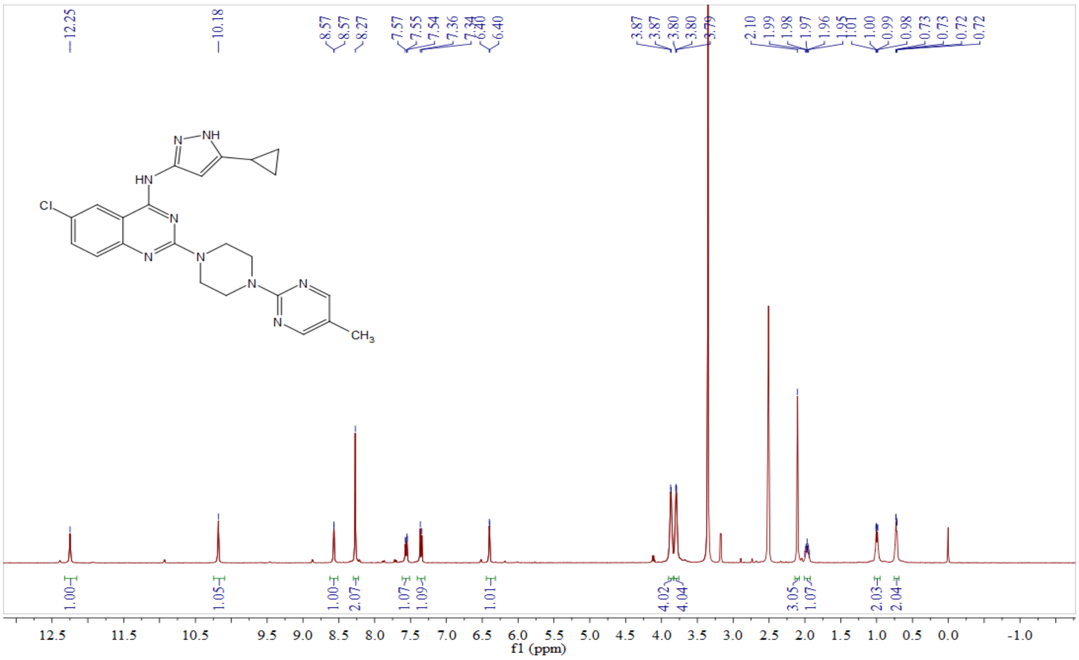
12.25
10.18
8.57 8.57 8.27
7.57 7.55 7.54 7.36 7.34 6.40 6.40
3.87 3.87 3.80 3.80 3.79
2.10 1.99 1.98 1.97 1.96 1.95 1.01 1.00 0.99 0.98 0.73 0.73 0.72 0.72
1.00 1.05 1.00 2.07 1.07 1.09 1.01 4.02 4.04 3.05 1.07 2.03 2.04
f1 (ppm)

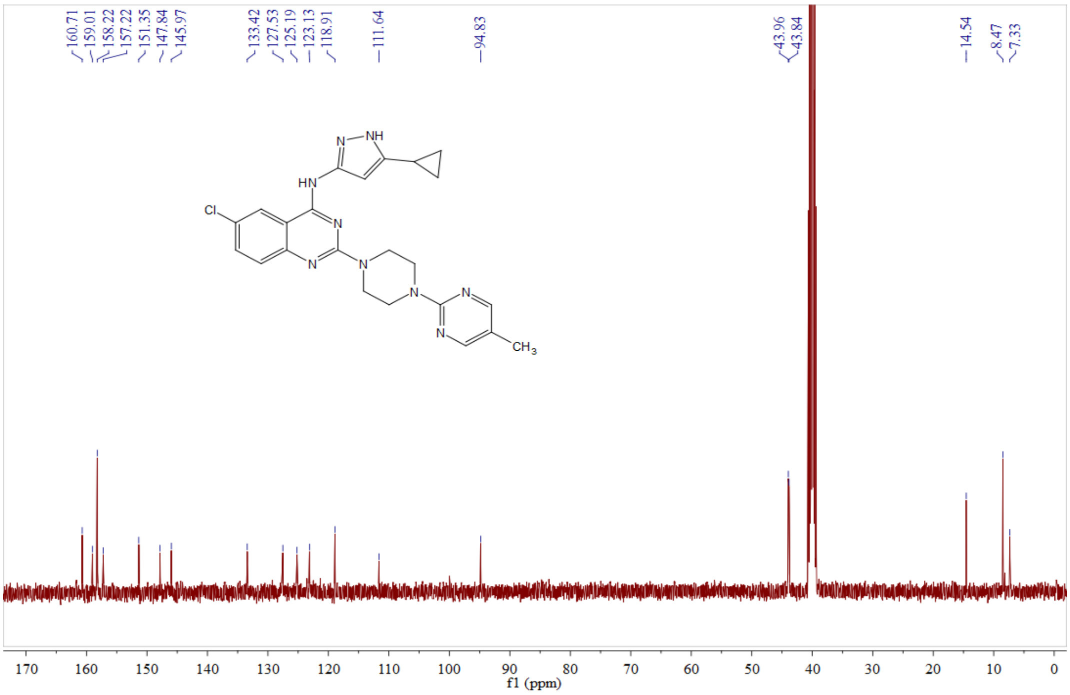
160.71 159.01 158.22 157.22 151.35 147.84 145.97
133.42 127.53 125.19 123.13 118.91
111.64
94.83
43.96 43.84
14.54
8.47 7.33
f1 (ppm)

User Spectra

Fragmentor Voltage	Collision Energy	Ionization Mode
200	0	ESI

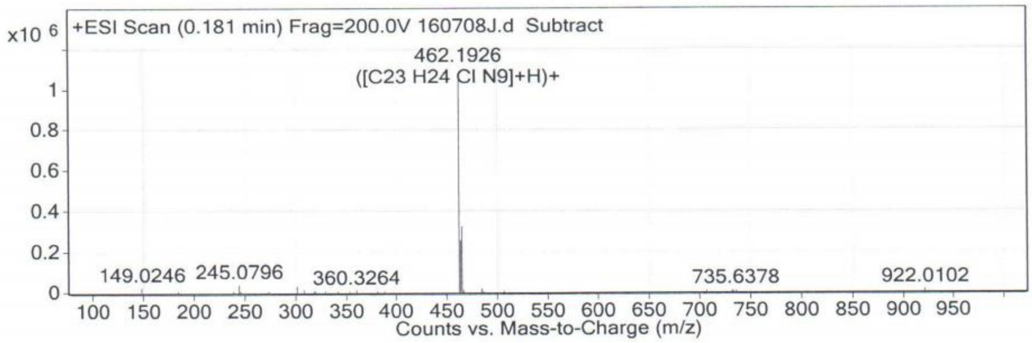

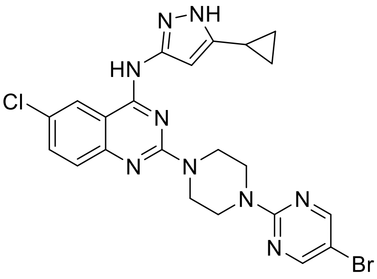

Compound **9c**

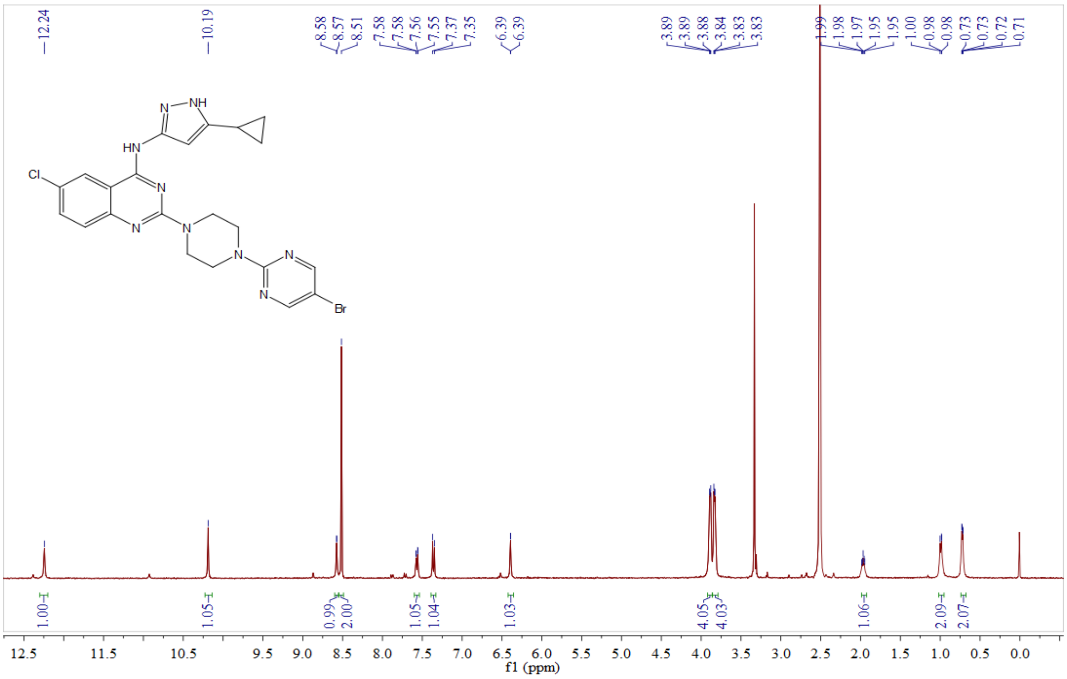
12.24
10.19
8.58
8.57
8.51
7.58
7.58
7.56
7.55
7.37
7.35
6.39
6.39
3.89
3.89
3.88
3.84
3.83
3.83
1.99
1.98
1.97
1.95
1.95
1.00
0.98
0.98
0.73
0.73
0.72
0.71
1.00
1.05
0.99
2.00
1.05
1.04
1.03
4.05
4.03
1.06
2.09
2.07
f1 (ppm)

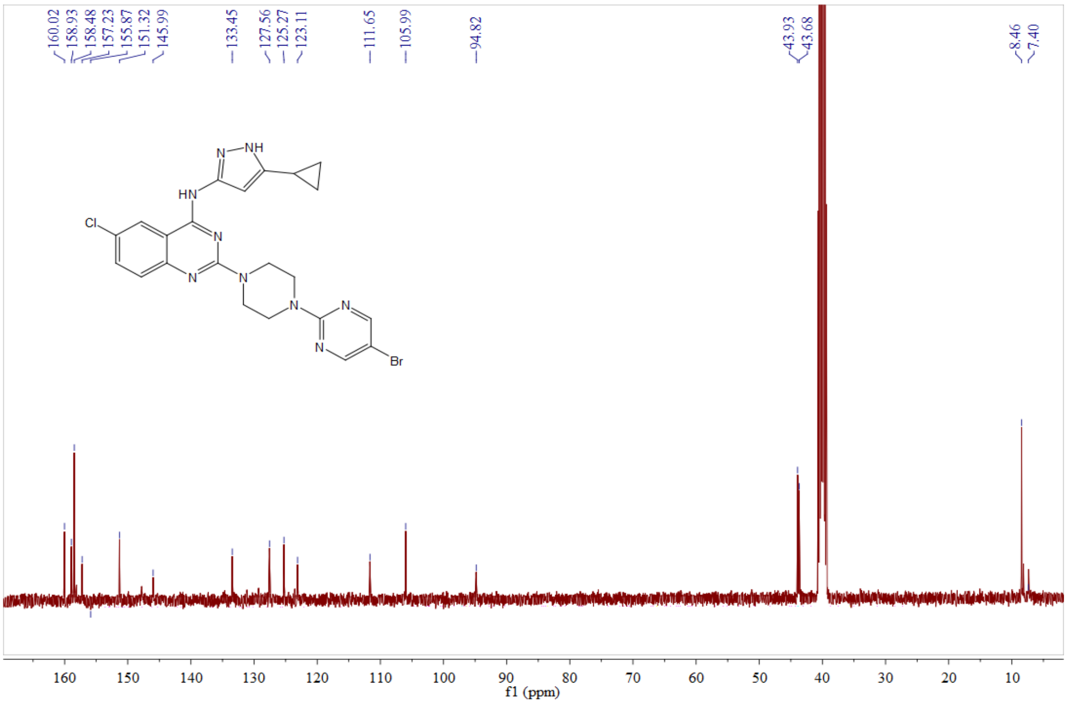
160.02
158.93
158.48
157.23
155.87
151.32
145.99
133.45
127.56
125.27
123.11
111.65
105.99
94.82
43.93
43.68
8.46
7.40
f1 (ppm)

User Spectra

Fragmentor Voltage	Collision Energy	Ionization Mode
200	0	ESI

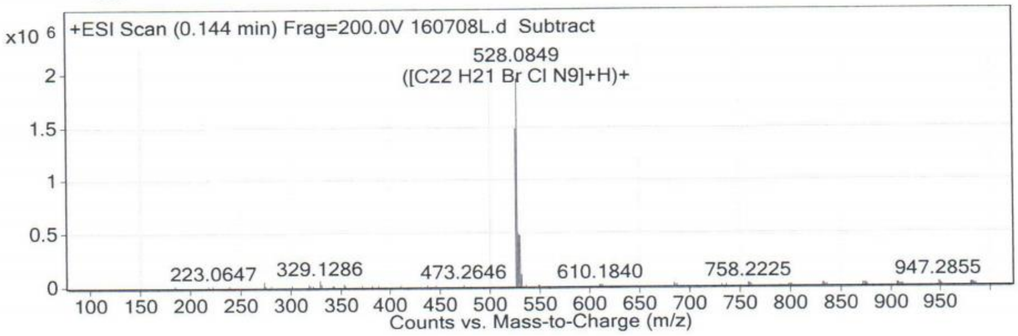

Supplement: Supplementary file 1 [file molecules-23-00417-s001.pdf]
